# Molecular characterization of AML‐MRC reveals *TP53* mutation as an adverse prognostic factor irrespective of MRC‐defining criteria, *TP53* allelic state, or *TP53* variant allele frequency

**DOI:** 10.1002/cam4.5421

**Published:** 2022-11-16

**Authors:** Davidson Zhao, Entsar Eladl, Mojgan Zarif, José‐Mario Capo‐Chichi, Andre Schuh, Eshetu Atenafu, Mark Minden, Hong Chang

**Affiliations:** ^1^ Department of Laboratory Medicine and Pathobiology University of Toronto Toronto Ontario Canada; ^2^ Department of Hematology and Medical Oncology University Health Network Toronto Ontario Canada; ^3^ Department of Pathology, Faculty of Medicine Mansoura University Mansoura Egypt; ^4^ Department of Biostatistics University Health Network Toronto Ontario Canada

**Keywords:** AML risk stratification, AML‐MRC, next‐generation sequencing, *TP53* mutation

## Abstract

**Background:**

Acute myeloid leukemia with myelodysplasia‐related changes (AML‐MRC) generally confers poor prognosis, however, patient outcomes are heterogeneous. The impact of *TP53* allelic state and variant allele frequency (VAF) in AML‐MRC remains poorly defined.

**Methods:**

We retrospectively evaluated 266 AML‐MRC patients who had NGS testing at our institution from 2014 to 2020 and analyzed their clinical outcomes based on clinicopathological features.

**Results:**

*TP53* mutations were associated with cytogenetic abnormalities in 5q, 7q, 17p, and complex karyotype. Prognostic evaluation of *TP53* ^MUT^ AML‐MRC revealed no difference in outcome between *TP53* double/multi‐hit state and single‐hit state. Patients with high *TP53* ^MUT^ variant allele frequency (VAF) had inferior outcomes compared to patients with low *TP53* ^MUT^ VAF. When compared to *TP53* ^WT^ patients, *TP53* ^MUT^ patients had inferior outcomes regardless of MRC‐defining criteria, *TP53* allelic state, or VAF. *TP53* mutations and elevated serum LDH were independent predictors for inferior OS and EFS, while *PHF6* mutations and transplantation were independent predictors for favorable OS and EFS. *NRAS* mutation was an independent predictor for favorable EFS.

**Conclusions:**

Our study suggests that *TP53* ^MUT^ AML‐MRC defines a very‐high‐risk subentity of AML in which novel therapies should be explored.

## Graphical abstract

*TP53*‐mutated AML‐MRC patients have inferior outcomes compared to *TP53*‐wildtype patients irrespective of MRC‐defining criteria, *TP53* allelic state or *TP53* variant allele frequency. *TP53*‐mutated AML‐MRC should be classified as a very‐high‐risk AML subentity for which novel therapeutic strategies should be explored.

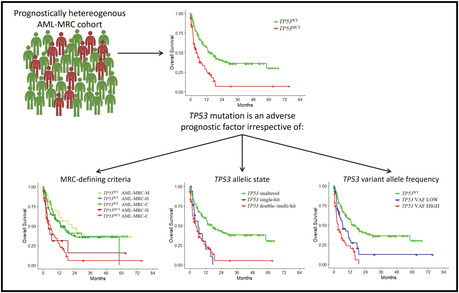

## 1 INTRODUCTION

Acute myeloid leukemia with myelodysplasia‐related changes (AML‐MRC) is a subentity of AML that accounts for approximately 25% of all AML cases. The diagnosis of AML‐MRC requires at least one of the following: (a) a prior history of myelodysplastic syndrome (MDS) or myelodysplastic/myeloproliferative neoplasm (MDS/MPN), (b) presence of a MDS‐defining cytogenetic abnormality, and (c) morphologic detection of multi‐lineage dysplasia (MLD). AML‐MRC is associated with advanced age, low remission rates, and poor prognosis, with a median overall survival of approximately 12 months.^1^, ^2^ Nonetheless, AML‐MRC patient outcomes remain heterogeneous.^2^, ^3^, ^4^, ^5^

In recent years, next‐generation sequencing (NGS) has become a standard tool to risk stratify patients with myeloid malignancies. In AML, mutations in *CEBPA*, *NPM1*, *FLT3*, *RUNX1*, *ASXL1*, and *TP53* have shown prognostic relevance and have been incorporated into the 2017 ELN risk stratification.^6^ However, there are limited studies that have described the molecular landscape of AML‐MRC and evaluated the prognostic impact of mutations within this subentity.

*TP53*, which is located on the short arm of chromosome 17, encodes the tumor suppressor protein p53 that mediates critical anti‐tumor activity by inducing apoptosis in response to DNA damage.^7^ *TP53* mutations are detected in up to 10% of de novo AML patients, however, its incidence has been shown to increase to approximately 30% in AML‐MRC.^8^, ^9^, ^10^ *TP53* mutations and abnormalities in chromosome 17p have been identified as poor prognostic factors by the 2017 ELN risk stratification and are associated with advanced age, therapy resistance, and poor prognosis.^6^, ^11^, ^12^ *TP53* mutations were recently identified to have an adverse prognostic impact in AML‐MRC.^5^ However, it remains unclear whether *TP53* allelic state and variant allele frequency (VAF) can further resolve the heterogeneity in AML‐MRC patient outcomes.

In the present study, we performed an extensive molecular evaluation of 266 AML‐MRC patients and retrospectively assessed whether individual mutations were associated with patient outcomes. We further characterized *TP53*‐mutated (*TP53* ^MUT^) AML‐MRC to evaluate whether *TP53* allelic state and clonal burden could resolve prognostic heterogeneity in AML‐MRC.

## 2 MATERIALS AND METHODS

### 2.1 Patients and therapy

We screened all the University Health Network patients diagnosed with AML‐MRC as per the 2016 WHO definition between April 2014 and November 2020. The study was approved by the University Health Network Research Ethics Board. Peripheral blood and bone marrow aspirate smears were reviewed by at least two independent hematopathologists and consensus on diagnosis was achieved. Patients were classified into three groups: (a) AML‐MRC patients with a history of MDS or of MDS/MPN, irrespective of the presence of MDS‐related cytogenetic changes (AML‐MRC‐H); (b) AML‐MRC patients with MDS‐defining cytogenetic abnormalities (AML‐MRC‐C); and (c) AML‐MRC patients with MLD alone, defined as the presence of >50% dysplasia in at least two lineages (AML‐MRC‐M). Patients with therapy‐related AML were excluded from this study. The majority of patients were treated with induction regimen consisting of cytarabine and daunorubicin/idarubicin, or non‐intensive regimens consisting of hypomethylating agent (HMA) or low‐dose cytarabine.

### 2.2 Karyotype analysis

In accordance with the International System for Human Cytogenetic Nomenclature guidelines, karyotypes were obtained from diagnostic bone marrow samples and described as appropriate. The cytogenetic loss of *TP53* was determined as previously described.^13^ Complex karyotype was defined as the presence of at least three cytogenetic abnormalities.

### 2.3 Mutational analysis and allocation of patients based on *TP53* allelic status

Next‐generation sequencing (NGS) was performed for 105 patients using a custom myeloid panel for 49 genes implicated in myeloid malignancies (Oxford Gene Technologies) and run on the MiSeq platform (Illumina), as previously described.^14^, ^15^ For 161 patients, NGS was performed using the TruSight Myeloid Sequencing Panel for 54 genes implicated in myeloid malignancies (Illumina) and run on the MiSeq platform (Illumina), as previously described.^16^, ^17^ The limit of detection for variant calling was 2%. On both panels, for 13/41 genes, the complete coding regions were sequenced, and for 28/41 genes, the same exonic hotspots were sequenced (Tables S1 and S2). Interpretation and classification of variants were performed as previously described.^15^ Variants detected by NGS are listed in Table S3. Of note, *TP53* mutations were assessed by NGS which spanned *TP53* exons 2–11 (Table S2), variants of unknown significance were excluded from analysis. When patients had multiple mutations in the same gene, the higher VAF was used for analysis.

Patients were considered to be double/multi‐hit *TP53* state when (A) at least two *TP53* variants were detected by NGS, (B) one *TP53* variant detected by NGS co‐occurred with cytogenetic loss of *TP53* and (C) one *TP53* variant was detected by NGS with a VAF of ≥55%, as previously described.^18^

### 2.4 Statistical analysis

Evaluation of patient outcomes including overall survival (OS) and event‐free survival (EFS) was carried out from retrospective analysis of patient records. OS was calculated as the time from the date of diagnosis to last follow‐up or death. EFS was defined as the time from the date of diagnosis to last follow‐up, relapse, or death. Univariate survival analysis and comparison of outcome were performed using the Kaplan–Meier product‐limit method and the log‐rank test. The chi‐square or Fisher's exact test, as appropriate, was used to assess associations between categorical parameters. The Kruskal–Wallis and Wilcoxon rank‐sum tests were used to assess whether there were differences in numerical variables between groups, as appropriate. Multivariable Cox proportional hazards regression was performed with variables that were significant prognostic factors by univariable analysis and variables that are known prognostic factors in AML. All statistical tests were performed using R software version 4.0.5 (R Core Team [2020]. R: A language and environment for statistical computing, Vienna, Austria) and were interpreted as significant if the two‐sided *p*‐value was less than 0.05.

## 3 RESULTS

### 3.1 Patient characteristics by MRC subtype and *TP53* mutation status

Two hundred and sixty‐six AML‐MRC patients were identified, including 142 (53.4%) AML‐MRC‐C patients, 99 (37.2%) AML‐MRC‐H patients, and 25 (9.4%) AML‐MRC‐M patients. The median age of our cohort was 70 years (range 18–91). The median follow‐up time was 6.2 months (range 0–75.7 months) and 160 (60%) patients died at the time of last follow‐up.

Patient characteristics as stratified by AML‐MRC subtype are summarized and compared in Table 1. Age, gender ratio, WBC count, platelet count, hemoglobin, serum LDH, and transplantation status were not significantly different across the three subtypes. Bone marrow blast % was elevated in AML‐MRC‐C compared to AML‐MRC‐H and AML‐MRC‐M (*p* = 0.032 and *p* = 0.018, respectively). The number of mutated genes was higher in AML‐MRC‐H and AML‐MRC‐M compared to AML‐MRC‐C (*p* = 0.00015 and *p* < 0.0001, respectively).

**TABLE 1:** Comparison of clinical features between AML‐MRC‐C, AML‐MRC‐H, and AML‐MRC‐M

Clinical feature	Total (*n* = 266)	AML‐MRC‐C (*n* = 142)	AML‐MRC‐H (*n* = 99)	AML‐MRC‐M (*n* = 25)	*p‐*value
Age (y), median [range]	70 [18–91]	70 [30–91]	70 [19–89]	73 [18–90]	0.475^a^
Male, *n* (%)	168 (63)	86 (61)	64 (65)	18 (72)	0.510^b^
WBC count ×10^9^/L, median [range]	3.4 [0.1–328.7]	3.4 [0.3–292.4]	3.7 [0.1–328.7]	3.2 [0.8–60.6]	0.606^a^
Platelets ×10^9^/L, median [range]	47 [3–1057]	47.5 [7–1057]	42 [3–703]	52.5 [14–490]	0.602^a^
Hemoglobin, g/dL, median [range]	85 [8–671]	85 [8–671]	87 [57–148]	82 [55–108]	0.452^a^
BM blasts %, median [range]	39.5 [20–95]	48 [20–95]	35 [20–91]	31 [20–82]	0.017^a^
LDH, IU/L, median [range]	304 [90–9473]	304 [94–9473]	305 [101–6550]	277 [90–1566]	0.535^a^
Number of mutated genes, median [range]	3 [0–9]	2 [0–8]	3 [0–9]	4 [1–7]	<0.0001^a^
Allo‐HSCT, *n* (%)	57 (21)	30 (21)	23 (23)	4 (16)	0.727^b^

Abbreviations: Allo‐HSCT, allogeneic hematopoietic stem cell transplantation; BM, bone marrow; LDH, lactate dehydrogenase; WBC, white blood cell.

^a^ Kruskal–Wallis test.

^b^ Chi‐square test.

Patient characteristics as stratified by *TP53* mutation status are summarized and compared in Table 2. Age, WBC count, platelet count, hemoglobin, bone marrow blast %, and serum LDH were not statistically different between *TP53* ^MUT^ and *TP53* ^WT^ patients. However, the *TP53* ^MUT^ group had a greater female representation and had fewer mutated genes other than *TP53* compared to the *TP53* ^WT^ group. Fewer *TP53* ^MUT^ patients had bone marrow transplantation.

**TABLE 2:** Comparison of clinical features between *TP53* ^MUT^ and *TP53* ^WT^ AML‐MRC

Clinical feature	Total (*n* = 266)	*TP53* ^MUT^ (*n* = 96)	*TP53* ^WT^ (*n* = 170)	*p‐*value
Age (y), median [range]	70 [18–91]	71.5 [38–91]	70 [18–90]	0.11^a^
Male, *n* (%)	168 (63)	51 (53)	117 (69)	0.016^b^
WBC count ×10^9^/L, median [range]	3.4 [0.1–328.7]	3.1 [0.1–76.9]	3.7 [0.5–328.7]	0.14^a^
Platelets ×10^9^/L, median [range]	47 [3–1057]	46 [3–355]	48 [7–1057]	0.078^a^
Hemoglobin, g/dL, median [range]	85 [8–671]	84 [57–671]	86 [8–169]	0.445^a^
BM blasts %, median [range]	39.5 [20–95]	38 [20–91]	40 [20–95]	0.493^a^
LDH, IU/L, median [range]	304 [90–9473]	320 [134–3569]	294 [90–9473]	0.11^a^
Number of non‐*TP53* mutated genes, median [range]	2 [0–9]	1 [0–4]	3 [0–9]	<0.0001^a^
Allo‐HSCT, *n* (%)	57 (21)	12 (13)	45 (26)	0.012^b^

Abbreviations: Allo‐HSCT, allogeneic hematopoietic stem cell transplantation; BM, bone marrow; LDH, lactate dehydrogenase; WBC, white blood cell.

^a^ Wilcoxon rank‐sum test.

^b^ Chi‐square test.

### 3.2 Mutation landscape of AML‐MRC

**FIGURE 1:**
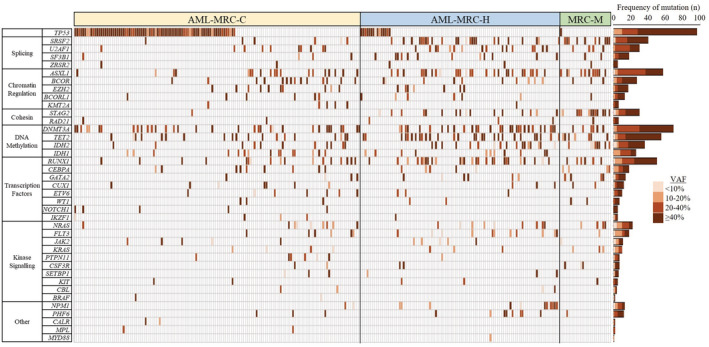
Co‐mutation plot for AML‐MRC patients. Mutations are colored by their variant allele frequency.

The mutational landscape of AML‐MRC is presented in Figure 1. The most frequently mutated gene was *TP53*, mutated in 96 (36%) patients, followed by *DNMT3A* (26%), *ASXL1* (21%), *TET2* (21%), *RUNX1* (19%), *SRSF2* (15%), *IDH2* (14%), *U2AF1* (11%), and *STAG2* (11%). 140 (53%) patients had at least one secondary‐type mutation involving *ASXL1*, *BCOR*, *EZH2*, *SF3B1*, *SRSF2*, *STAG2*, *U2AF1*, and *ZRSR2*, as defined by Lindsley et al.^19^

The frequency of mutations in each AML‐MRC subtype is presented in Figure S1 and an association analysis of the frequently mutated genes is described here. *TP53* mutations were significantly enriched in AML‐MRC‐C compared to both AML‐MRC‐H and AML‐MRC‐M (*p* < 0.0001 and *p* < 0.0001, respectively). *STAG2* mutations were significantly enriched in AML‐MRC‐M compared to both AML‐MRC‐C and AML‐MRC‐H (*p* < 0.0001 and *p* = 0.0004, respectively), and were significantly enriched in AML‐MRC‐H compared to AML‐MRC‐C (*p* < 0.0001). Mutations significantly enriched in both AML‐MRC‐H and AML‐MRC‐M relative to AML‐MRC‐C were in *ASXL1* (*p* = 0.011 and *p* = 0.0008, respectively), *SRSF2* (*p* < 0.0001 and *p* < 0.0001, respectively) and *IDH2* (*p* = 0.041 and *p* = 0.0021, respectively). Mutations in *U2AF1*, *RUNX1* and *TET2* were significantly enriched only in AML‐MRC‐H compared to AML‐MRC‐C (*p* = 0.042, *p* = 0.0043 and *p* = 0.0040, respectively). Overall, AML‐MRC‐C was characterized by *TP53* mutations, while AML‐MRC‐H and AML‐MRC‐M were characterized by secondary‐type mutations. Indeed, secondary‐type mutations were more frequently harbored in AML‐MRC‐M (21/25 patients, 84%) and AML‐MRC‐H (67/99 patients, 68%) compared to AML‐MRC‐C (52/142 patients, 37%) (*p* < 0.0001 and *p* < 0.0001, respectively).

### 3.3 Mutational complementation groups in AML‐MRC

Molecular characterization of AML‐MRC allowed us to identify frequently co‐occurring and mutually exclusive mutations in AML‐MRC. Our correlation analysis of recurrently (≥5) mutated genes is presented in Figure S2. Mutations in *ASXL1*, *RUNX1*, *SRSF2*, and *IDH2* were significantly underrepresented in the *TP53* ^MUT^ group. *ASXL1* mutations significantly co‐occurred with mutations in *RUNX1*, *SRSF2*, *IDH2*, and *STAG2*. *STAG2* mutations also significantly co‐occurred with mutations in *SRSF2*, *IDH2*, and *NRAS*. Other significantly co‐occurring mutation pairs included *IDH2* and *SRSF2*, *IDH2* and *FLT3*, and *U2AF1* and *KRAS*.

### 3.4 Association of *TP53* mutations with cytogenetic abnormalities

Karyotype analysis was available in 248 (93%) AML‐MRC patients, including 92 *TP53* ^MUT^ and 156 *TP53* ^WT^ patients. *TP53* ^MUT^ patients were more likely to have complex karyotype than *TP53* ^WT^ patients (89/92 patients, 97% vs. 41/156 patients, 26%, respectively, *p* < 0.0001). *TP53* ^MUT^ patients were also more likely to have cytogenetic abnormalities in chromosome 17p (41/92 patients, 45% vs. 7/156 patients, 5%, respectively, *p* < 0.0001).

Among the 18 patients without available karyotype, 12 patients had interphase FISH for del(5q) and del(7q). *TP53* ^MUT^ patients were more likely to have chromosomal abnormalities in 5q (71/95 patients, 75% vs. 23/165 patients, 14%, respectively, *p* < 0.0001) and 7q (54/95 patients, 57% vs. 38/165, 23%, respectively, *p* < 0.0001).

Overall, chromosomal aberrations in 17p had the highest specificity (95%) for *TP53* mutations, and complex karyotype had the highest sensitivity (97%) for *TP53* mutations.

### 3.5 Outcomes of AML‐MRC patients based on subtype and genetic features

The median OS and EFS of our cohort were 10.7 months and 7.7 months, respectively. Kaplan–Meier curves for patients as stratified by subtype are presented in Figure S3A,B. AML‐MRC‐M patients had borderline significantly better OS (HR 0.59, 95% CI 0.34–1.02, *p* = 0.058) and had significantly better EFS (HR 0.58, 95% CI 0.35–0.97, *p* = 0.037) compared to AML‐MRC‐C patients. AML‐MRC‐H patients had borderline significantly better OS (HR 0.74, 95% CI 0.53–1.04, *p* = 0.081) and EFS (HR 0.75, 95% CI 0.54–1.02, *p* = 0.068) compared to AML‐MRC‐C patients. AML‐MRC‐M patients had no significant differences in OS (HR 0.78, 95% CI 0.44–1.39, *p* = 0.40) and EFS (HR 0.77, 95% CI 0.45‐1.32, *p* = 0.34) compared to AML‐MRC‐H patients.

**FIGURE 2:**
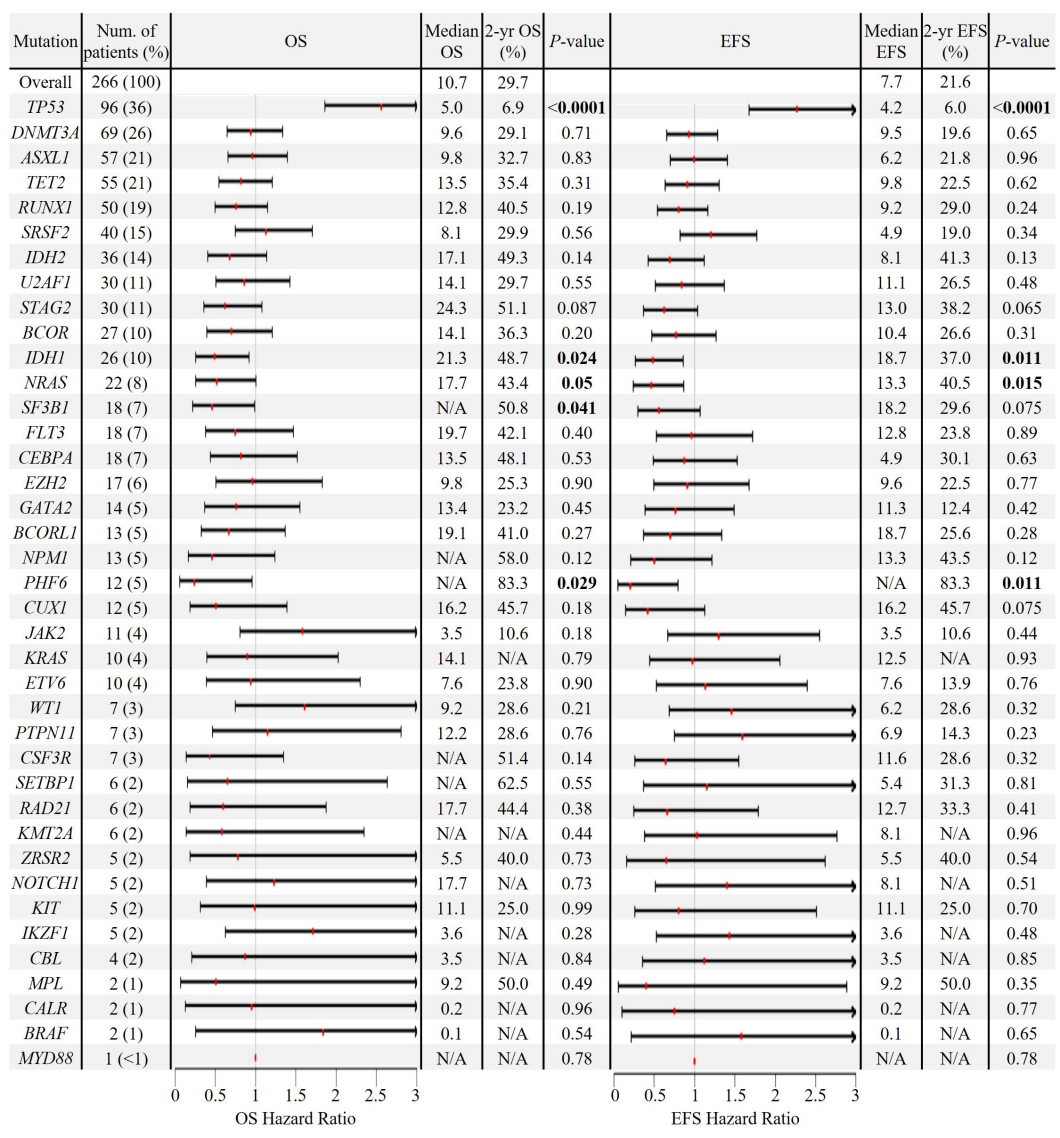
Forest plot indicating the OS and EFS hazard ratio and 95% confidence interval of mutations in AML‐MRC. Median and 2‐year survival and log‐rank *p*‐value are listed to the right.

Given the paucity of data on the prognostic impact of mutations in AML‐MRC, we evaluated the associations between individual gene mutations and patient outcomes. The OS and EFS hazard ratios, median and 2‐year OS and EFS, and log‐rank *p*‐values for all mutations are presented in Figure 2. *TP53* mutations were associated with inferior OS and EFS, while mutations in *IDH1*, *NRAS*, and *PHF6* were associated with favorable OS and EFS. *SF3B1* mutations were associated with favorable OS but not EFS. The respective Kaplan–Meier curves are provided in Figures S4 and S5.

**FIGURE 3:**
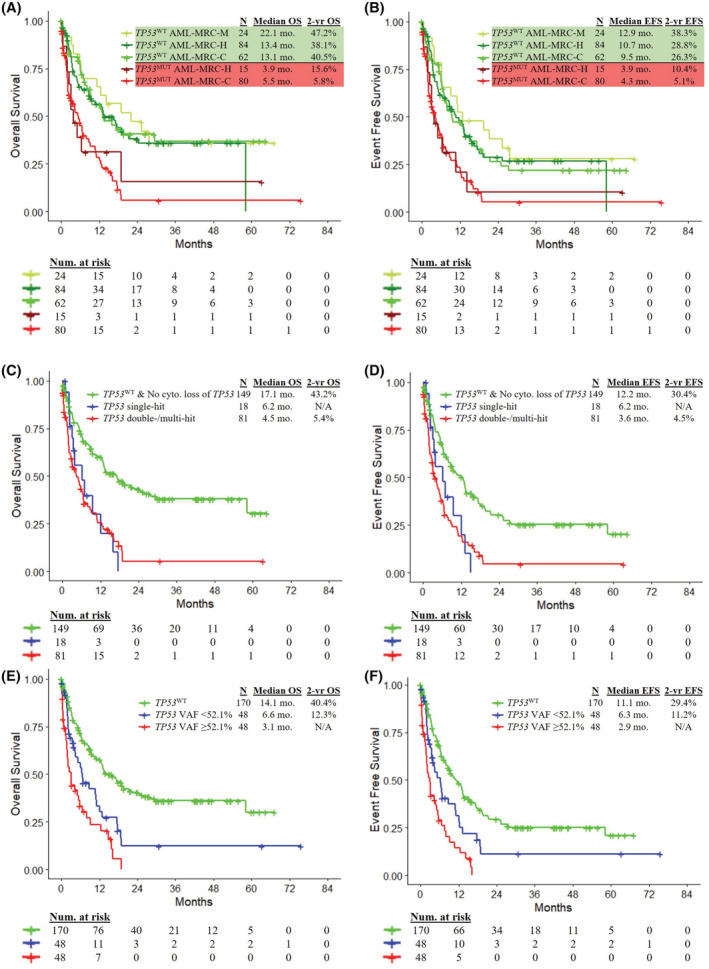
Kaplan–Meier estimates for OS and EFS of AML‐MRC patients stratified by (A, B) AML‐MRC subtype and *TP53* mutation status, (C, D) number of hits to *TP53*, (E, F) median *TP53* variant allele frequency (VAF).

Kaplan–Meier curves for patients as stratified by both subtype and *TP53* mutation status are presented in Figure 3A,B. *TP53* mutations were detected in 80 (56%) AML‐MRC‐C patients, 15 (15%) AML‐MRC‐H patients, and one (4%) AML‐MRC‐M patient. *TP53* mutation had an adverse impact on OS and EFS in AML‐MRC‐C (HR 2.52, 95% CI 1.62–3.93, *p* < 0.0001 and HR 2.14, 95% CI 1.42–3.21, *p* = 0.0002, respectively). *TP53* mutation was associated with borderline significantly inferior OS (HR 1.93, 95% CI 0.98–3.78, *p* = 0.053) and significantly inferior EFS (HR 1.89, 95% CI 0.99–3.59, *p* = 0.049, respectively) in AML‐MRC‐H. For both *TP53* ^MUT^ and *TP53* ^WT^ patients, AML‐MRC subtype had no significant impact on OS (*TP53* ^MUT^: *p* = 0.39; *TP53* ^WT^: *p* = 0.79) or EFS (*TP53* ^MUT^: *p* = 0.43; *TP53* ^WT^: *p* = 0.68).

Cytogenetic loss of *TP53* was associated with inferior OS (HR 2.49, 95% CI 1.70–3.63, *p* < 0.0001) and EFS (HR 2.25, 95% CI 1.57–3.23, *p* < 0.0001) (Figure S6A,B). Among patients with *TP53* ^MUT^, additional *TP53* variant(s) were detected in 26 patients and did not significantly impact OS (HR 0.68, 95% CI 0.39–1.17, *p* = 0.16) or EFS (HR 0.65, 95% CI 0.38–1.11, *p* = 0.11) (Figure S6C,D). Compared to *TP53* ^WT^ patients, *TP53* ^MUT^ patients with one *TP53* mutation had inferior OS (HR 2.79, 95% CI 1.96–3.96, *p* < 0.0001) and EFS (HR 2.56, 95% CI 1.83–3.57, *p* < 0.0001), and *TP53* ^MUT^ patients with multiple *TP53* mutations had inferior OS (HR 1.89, 95% CI 1.12–3.19, *p* = 0.015) and borderline significantly inferior EFS (HR 1.63, 95% CI 0.99–2.69, *p* = 0.054) (Figure S6C,D). Among patients with *TP53* ^MUT^, concurrent cytogenetic loss of *TP53* did not significantly impact OS (HR 1.35, 95% CI 0.83–2.20, *p* = 0.22) or EFS (HR 1.31, 95% CI 0.82–2.10, *p* = 0.25) (Figure S6E,F). Compared to unaltered *TP53* patients, all altered *TP53* patients, either with cytogenetic loss of *TP53* alone, *TP53* mutation alone, or *TP53* mutation with concurrent cytogenetic loss of *TP53* had inferior OS (HR 2.90, 95% CI 1.32–6.36, *p* = 0.0055; HR 2.36, 95% CI 1.58–3.52, *p* < 0.0001; HR 3.14, 95% CI 2.03–4.84, *p* < 0.0001, respectively) and EFS (HR 2.34, 95% CI 1.08–5.10, *p* = 0.027; HR 2.07, 95% CI 1.42–3.04, *p* < 0.0001; HR 2.81, 95% CI 1.86–4.24, *p* < 0.0001, respectively) (Figure S6E,F).

A workflow to allocate *TP53* allelic state in our cohort is presented in Figure S7. AML‐MRC patients with *TP53* double/multi‐hit had no significant differences in OS (HR 1.10, 95% CI 0.60–2.01, *p* = 0.76) and EFS (HR 1.20, 95% CI 0.66–2.19, *p* = 0.55) compared to patients with *TP53* single‐hit (Figure 3C,D). Compared to unaltered *TP53* patients, both *TP53* single‐hit and *TP53* double/multi‐hit patients had inferior OS (HR 2.48, 95% CI 1.35–4.54, *p* = 0.0023 and HR 2.73, 95% CI 1.93–3.85, *p* < 0.0001, respectively) and EFS (HR 2.08, 95% CI 1.15–3.76, *p* = 0.014 and HR 2.40, 95% CI 1.73–3.31, *p* < 0.0001, respectively) (Figure 3C,D).

When stratified by the median *TP53* ^MUT^ VAF (52.1%), patients with high VAF had inferior OS (HR 1.75, 95% CI 1.09–2.82, *p* = 0.019) and EFS (HR 1.90, 95% CI 1.18–3.05, *p* = 0.0068) compared to low VAF patients (Figure 3E,F). Compared to *TP53* ^WT^ patients, all *TP53* ^MUT^ patients with either low or high *TP53* ^MUT^ VAF had inferior OS (HR 1.93, 95% CI 1.29–2.89, *p* = 0.0012 and HR 3.38, 95% CI 2.26–5.05, *p* < 0.0001, respectively) and EFS (HR 1.67, 95% CI 1.13–2.47, *p* = 0.0089 and HR 3.20, 95% CI 2.19–4.68, *p* < 0.0001, respectively) (Figure 3E,F). Similar results were observed with *TP53* ^MUT^ VAF threshold of 40%: AML‐MRC patients with high VAF had borderline significantly inferior OS (HR 1.59, 95% CI 0.93–2.73, *p* = 0.086) and EFS (HR 1.67, 95% CI 0.99–2.82, *p* = 0.053) compared to low VAF patients (Figure S8A,B). Compared to *TP53* ^WT^ patients, all AML‐MRC patients with either *TP53* ^MUT^ VAF <40% or ≥40% had inferior OS (HR 1.84, 95% CI 1.10–3.08, *p* = 0.018 and HR 2.81, 95% CI 1.98–4.00, *p* < 0.0001, respectively) and EFS (HR 1.63, 95% CI 1.00–2.67, *p* = 0.050 and HR 2.56, 95% CI 1.83–3.57, *p* < 0.0001, respectively) (Figure S8A,B).

In *TP53* ^MUT^ AML‐MRC patients, concurrent *DNMT3A* mutation was associated with inferior OS (HR 2.12, 95% CI 1.20–3.76, *p* = 0.0083) and EFS (HR 1.96, 95% CI 1.11–3.45, *p* = 0.018), while in *TP53* ^WT^ patients, *DNMT3A* mutation did not significantly impact OS (HR 0.81, 95% CI 0.51–1.30, *p* = 0.39) or EFS (HR 0.84, 95% CI 0.55–1.28, *p* = 0.41) (Figure S9A,B).

Interestingly, AML‐MRC patients with ≥6 mutated genes had better OS (HR 0.46, 95% CI 0.23–0.90, *p* = 0.02) and EFS (HR 0.50, 95% CI 0.27–0.92, *p* = 0.024) compared to patients with <6 mutated genes (Figure S10A,B). Similarly, when using a threshold of three mutations, patients with higher number of mutated genes had borderline significantly better OS (HR 0.74, 95% CI 0.54–1.01, *p* = 0.055) and EFS (HR 0.78, 95% CI 0.58–1.05, *p* = 0.097) (Figure S10C,D).

Both *TP53* ^WT^ and *TP53* ^MUT^ patients who went onto transplant had significantly longer OS (HR 0.13, 95% CI 0.06–0.28, *p* < 0.0001 and HR 0.37, 95% CI 0.17–0.77, *p* = 0.0063, respectively) and EFS (HR 0.31, 95% CI 0.19–0.52, *p* < 0.0001 and HR 0.44, 95% CI 0.22–0.87, *p* = 0.015, respectively) than those who did not, however, *TP53* ^WT^ patients had dramatically improved and plateauing long‐term survival rates (Figure S11A–D).

By multivariable analysis for OS, *TP53* mutation and elevated serum LDH levels were independent predictors for adverse outcomes, while *PHF6* mutation and transplantation were independently associated with improved outcomes (Table 3). For EFS, *TP53* mutation and elevated serum LDH levels were independent predictors for adverse outcomes, while *NRAS* mutation, *PHF6* mutation, and transplantation were independently associated with improved outcomes (Table 3).

**TABLE 3:** Multivariable analysis for overall survival and event‐free survival

Characteristic	Overall survival		Event free survival	
	HR (95% CI)	*P*‐value	HR (95% CI)	*p*‐value
*TP53* mutation	1.77 (1.17–2.67)	0.007	1.60 (1.10–2.33)	0.013
*SF3B1* mutation	0.50 (0.23–1.09)	0.082	—	—
*IDH1* mutation	0.81 (0.42–1.58)	0.539	0.69 (0.38–1.27)	0.239
*PHF6* mutation	0.15 (0.03–0.87)	0.034	0.15 (0.03–0.78)	0.024
*NRAS* mutation	0.58 (0.28–1.19)	0.136	0.49 (0.25–0.96)	0.039
Age^a^	1.00 (0.99–1.02)	0.865	1.00 (0.99–1.01)	0.876
WBC count^a^	1.00 (0.99–1.00)	0.936	1.00 (1.00–1.00)	0.971
LDH^a^	1.00 (1.00–1.00)	0.0010	1.00 (1.00–1.00)	0.0013
AML‐MRC subtype				
AML‐MRC‐M	Reference	—	—	—
AML‐MRC‐C	1.38 (0.74–2.59)	0.312	1.48 (0.83–2.63)	0.182
AML‐MRC‐H	1.37 (0.76–2.45)	0.295	1.35 (0.78–2.33)	0.283
Allo‐HSCT	0.21 (0.12–0.37)	<0.0001	0.37 (0.24–0.58)	<0.0001

Abbreviations: Allo‐HSCT, allogeneic hematopoietic stem cell transplantation; LDH, lactate dehydrogenase; WBC, white blood cell.

^a^ Age, WBC count, and LDH were analyzed as continuous variables.

## 4 DISCUSSION

In this study, we report, to the best of our knowledge, the largest molecular profiling of AML‐MRC by detailed NGS. We provide a comprehensive evaluation of the prognostic impact of commonly occurring myeloid gene mutations in AML‐MRC. We identified *TP53*, *PHF6*, and *NRAS* mutations to be independent predictors of outcome. In addition, we provide an in‐depth characterization and prognostic evaluation of *TP53* ^MUT^ AML‐MRC, revealing that (A) the overall adverse prognosis in AML‐MRC is largely driven by *TP53* mutation status and not AML‐MRC‐defining criteria, and (B) *TP53* mutation confers an adverse prognostic impact in AML‐MRC irrespective of its allelic state or VAF.

Recent advances in molecular analysis has greatly improved AML risk stratification and classification. The 2022 WHO classification of AML has incorporated genomic alterations in *NPM1*, *CEBPA*, and secondary‐type mutations in *ASXL1*, *BCOR*, *EZH2*, *SF3B1*, *SRSF2*, *STAG2*, *U2AF1*, and *ZRSR2* owing to their association with distinct clinical features and outcomes.^20^ However, the 2022 WHO classification still relies heavily on cytogenetic alterations to define AML subentities and does not consider *TP53* mutations to define a standalone AML subtype.^20^ In contrast, the new 2022 International Consensus Classification of Myeloid Neoplasms and Acute Leukemias recognizes “AML with mutated *TP53*” as a distinct disease category owing to its association with distinctly aggressive disease, complex cytogenetic abnormalities and very poor outcome.^21^ In our cohort, *TP53* mutations were independently associated with adverse outcomes in AML‐MRC. Strikingly, when stratifying patients by both AML‐MRC subtype and *TP53* mutation status, patient outcomes largely grouped together by *TP53* mutation status, not AML‐MRC subtype (Figure 3A,B). This suggests that it is the underlying biology of *TP53* mutations rather than AML‐MRC criteria such as MDS‐defining cytogenetic abnormalities that confer adverse outcomes in AML. With the emergence of new therapeutic agents that target specific genetic alterations, there is a strong rationale for a genomics‐based AML classification system.

To date, frontline therapy for AML‐MRC has remained unaltered over the past 40+ years as induction with cytarabine and daunorubicin, followed by transplantation in complete remission. The recently approved CPX‐351 liposomal formulation of cytarabine and daunorubicin was shown to significantly improve secondary AML patient outcomes compared to conventional 7 + 3.^22^, ^23^, ^24^ However, *TP53* ^MUT^ AML patients treated with CPX‐351 or other chemotherapy regimens have adverse outcomes.^25^, ^26^, ^27^, ^28^, ^29^, ^30^ In addition, studies have shown that *TP53* ^MUT^ patients who go on to transplant have dismal long‐term outcomes, which is consistent with our data that show *TP53* ^WT^ patients and not *TP53* ^MUT^ patients had dramatically improved outcomes and plateauing long‐term survival rates after transplantation.^31^, ^32^ While the treatment of *TP53* ^MUT^ remains a challenge, clinical trials for *TP53* ^MUT^ AML using targeted therapies and immunotherapies have shown great potential.^33^, ^34^, ^35^, ^36^, ^37^, ^38^, ^39^

With the emergence of clinical trials for *TP53* ^MUT^ AML patients, there is an increasing need to timely detect *TP53* mutations at the start of treatment. The introduction of NGS covering the prognostically relevant 2017 ELN mutations has largely negated the necessity of more specific molecular tests such as PCR or FISH. To date, *TP53* mutation is commonly assessed only by NGS, including at our institution. However, with the slow turnaround times for NGS, physicians might not be able to identify *TP53* ^MUT^ AML patients at the start of treatment, thus leading to suboptimal therapeutic decisions. Therefore, faster turnaround tests for *TP53* mutation should be further explored in the clinical setting and implemented into the standard AML patient workup. However, as we show here, chromosomal aberrations in 5q, 7q, 17p, and complex karyotype may serve as surrogate markers for *TP53* mutations.

The prognostic impact of *TP53* allelic state in AML remains controversial. As a tumor suppressor gene, it is conventionally thought that *TP53* alterations should have a pathogenic effect only in the bi‐allelic or “double‐hit” setting. In MDS, bi‐allelic and not mono‐allelic *TP53* alterations have been shown to predict for distinctly adverse outcomes and poor response to therapy,^40^, ^41^ thus “MDS with bi‐allelic *TP53* alteration” has been proposed as a provisional entity in the upcoming 5th edition of the WHO classification of myeloid neoplasms and acute leukemia.^42^ In AML, the prognostic impact of *TP53* allelic state remains controversial and studies have reported conflicting data.^5^, ^41^, ^43^, ^44^ Interestingly, molecular analysis of serial patient samples has provided evidence that AML therapy induces evolutionary pressures for loss‐of‐heterozygosity and expansion of newly acquired double‐hit *TP53* cell populations^45^, ^46^ Thus, AML patients initially presenting with mono‐allelic *TP53* alteration may have similar clinical outcomes as patients with bi‐allelic *TP53* alteration. Here, we report that *TP53* allelic state does not have prognostic implication in AML‐MRC. No significant difference in outcome was observed in AML‐MRC patients when (A) stratifying patients by number of *TP53* mutations, (B) stratifying *TP53* ^MUT^ patients by the presence/absence of cytogenetic loss of *TP53*, or (C) stratifying patients by the number of *TP53* hits. Our data suggest that AML‐MRC with any number of *TP53* hits should be considered as very‐high‐risk AML.

The association between *TP53* clonal burden and AML patient outcomes remains controversial. Prior studies have reported conflicting data on the prognostic impact of *TP53* ^MUT^ VAF in both non‐intensive and induction chemotherapy patients.^18^, ^43^, ^47^, ^48^, ^49^ Here, we report that AML‐MRC patients with high *TP53* ^MUT^ VAF had inferior OS and EFS compared to patients with low VAF. Therefore, AML‐MRC patients with high *TP53* ^MUT^ VAF may benefit most from novel *TP53* ^MUT^ therapies. However, low *TP53* ^MUT^ VAF was also associated with poor outcomes and should still be considered as a high‐risk factor for AML‐MRC.

It is conventionally thought that greater mutational complexity predicts for inferior outcomes due to clonal heterogeneity. Prior studies have reported associations between higher number of mutations and inferior outcomes in AML.^8^, ^50^ Interestingly, we showed that in AML‐MRC, higher number of mutated genes was associated with favorable outcomes. *TP53* mutations, which independently predicted for poor outcomes in AML‐MRC, were infrequently accompanied by co‐occurring mutations, which was consistent with a prior report.^51^ As such, the discrepancy in the prognostic impact of mutational complexity in AML overall and AML‐MRC may be partly explained by the higher frequency of *TP53* mutations in AML‐MRC.

In addition to identifying *TP53* mutations as having independent prognostic value, we also showed that *NRAS* and *PHF6* mutations were independent predictors for improved outcomes. Even after exclusion of AML‐MRC‐M cases, *PHF6* mutations retained their association with improved OS (HR 0.06, 95% CI 0.006–0.69, *p* = 0.024) and EFS (HR 0.06, 95% CI 0.006–0.65, *p* = 0.020), while *NRAS* mutations retained their association with improved EFS (HR 0.44, 95% CI 0.21–0.91, *p* = 0.028) in multivariable analysis (data not shown). *NRAS* mutations were reported to have a favorable prognostic effect in *NPM1* ^MUT^/*DNMT3A* ^MUT^ AML.^8^ While *NRAS* mutations have not shown a significant prognostic impact in AML overall, studies have shown that *NRAS‐*mutated clones are chemosensitive and do not persist after treatment.^52^, ^53^, ^54^, ^55^, ^56^ In contrast, there are limited data on the prognostic impact of *PHF6* mutations in AML, likely due to its low frequency (2%–3%) in AML.^57^ Patel et al^58^ reported that *PHF6* mutations were associated with adverse outcomes in AML overall and intermediate‐risk AML. Another study in CK‐AML patients revealed that when stratifying patients into “typical CK”, defined as CK containing high‐risk abnormalities in 5q, 7q, and/or 17p, and “atypical CK”, defined as CK without such abnormalities, CK‐AML patients with atypical CK had a significantly higher rate of *PHF6* mutation and favorable outcomes.^59^ Here, we show for the first time that *NRAS* and *PHF6* mutations have an independently favorable prognostic impact in AML‐MRC. Future studies are needed to evaluate whether *NRAS* ^MUT^ or *PHF6* ^MUT^ AML‐MRC should still be classified as high‐risk AML.

We acknowledge that our study has several limitations. First, our study is limited by its retrospective nature and its relatively small subsets for analysis. Future prospective studies with larger cohorts are needed to confirm our data. Second, NGS was performed with two different gene panels which may have resulted in variability in the reported VAFs due to technical differences. However, all NGS experiments were performed on the same MiSeq platform. Third, our *TP53* ^MUT^ VAF threshold of the median may not reflect a biologically meaningful threshold, however, this also applies to other prognostic biomarkers such as WBC count. Future investigations are needed to optimize the *TP53* ^MUT^ VAF threshold for risk stratification.

## 5 CONCLUSION

In conclusion, *TP53* ^MUT^ AML‐MRC patients had inferior outcomes compared to *TP53* ^WT^ patients irrespective of MRC‐defining criteria, *TP53* allelic state, or *TP53* VAF. Our study suggests that *TP53* ^MUT^ AML‐MRC should be classified as a very‐high‐risk AML subentity and highlights the great need for novel therapies for *TP53* ^MUT^ AML.

## AUTHORS’ CONTRIBUTIONS

DZ analyzed data, performed statistical analysis, and wrote the manuscript. DZ, EE, MZ, JM, AS and MM collected data for analysis. EA performed statistical analysis. HC designed the study, analyzed data, supervised the project, and wrote the manuscript.

## FUNDING INFORMATION

This research was supported by grants from the Leukemia and Lymphoma Research Society of Canada (LLSC) and the Cancer Research Society (CRS).

## CONFLICT OF INTEREST

The authors declare no conflicts of interest.

## ETHICS APPROVAL STATEMENT

The study was approved by the UHN Research Ethics Board.

## Supporting information


Data S1

## Data Availability

Data are available on request from the corresponding author.
