# Supplementary material for: Molecular characterization of AML‐MRC reveals TP53 mutation as an adverse prognostic factor irrespective of MRC‐defining criteria, TP53 allelic state, or TP53 variant allele frequency
Source: Cancer Med. 2022 Nov 16;12(6):6511–22. doi: 10.1002/cam4.5421 (PMC10067127; doi:10.1002/cam4.5421)
Supplement: Supplementary file 1 — Data S1 [file CAM4-12-6511-s001.docx]

**Supplementary Data**

**Figure S1.** Frequency of individual mutations in 142 AML-MRC-C patients, 99 AML-MRC-H patients and 25 AML-MRC-M patients. Bars are colored based on the AML-MRC subtype.

**
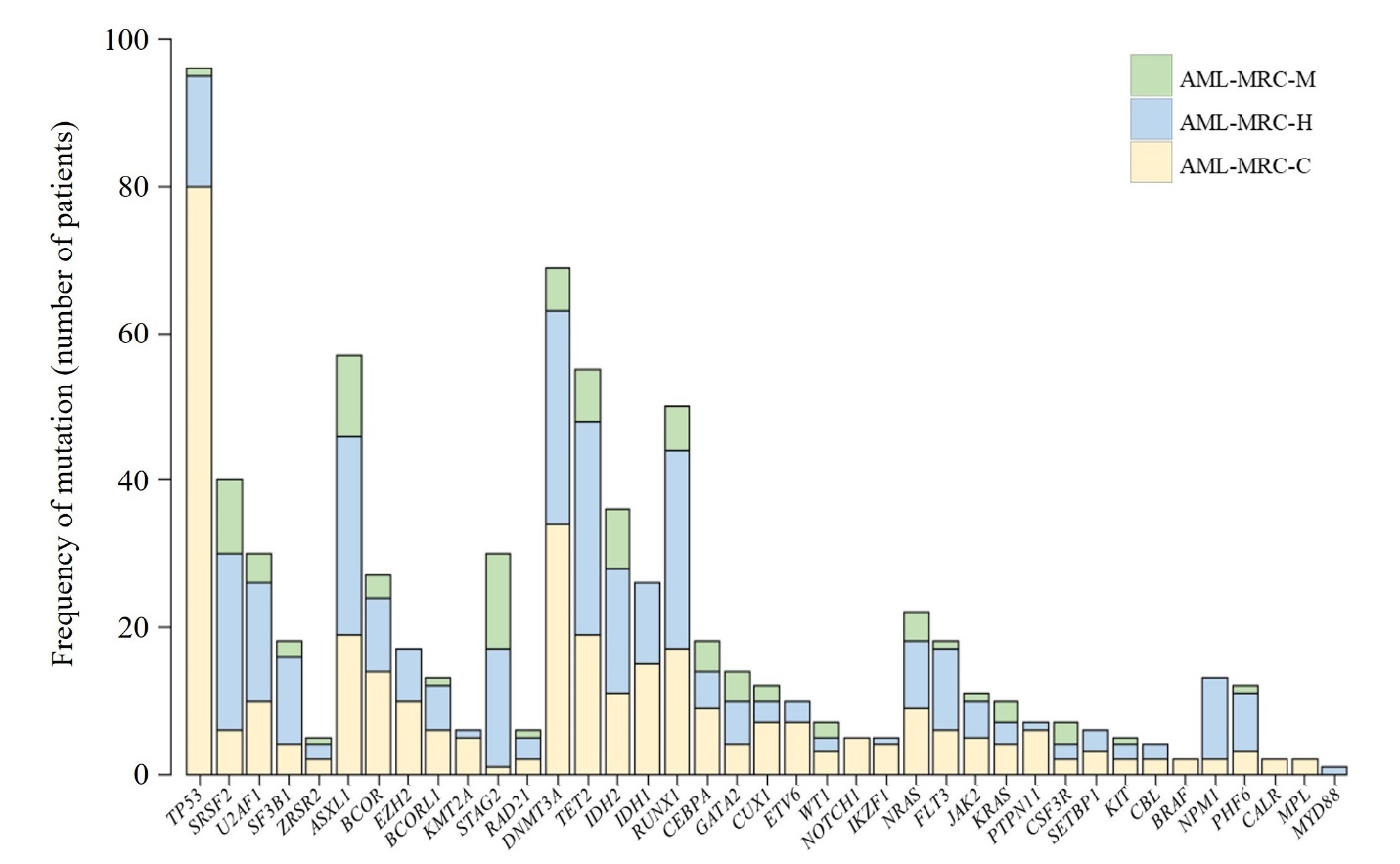
**

**Figure S2.** Pearson correlation coefficient matrix for recurrent mutations (≥5) in AML-MRC. Boxes are coloured based on correlation strength and direction of relationship. Dark red represents a strong positive correlation and dark blue represents a strong negative correlation. An underline under the respective correlation coefficients indicates P<0.05 after FDR correction for multiple comparisons.


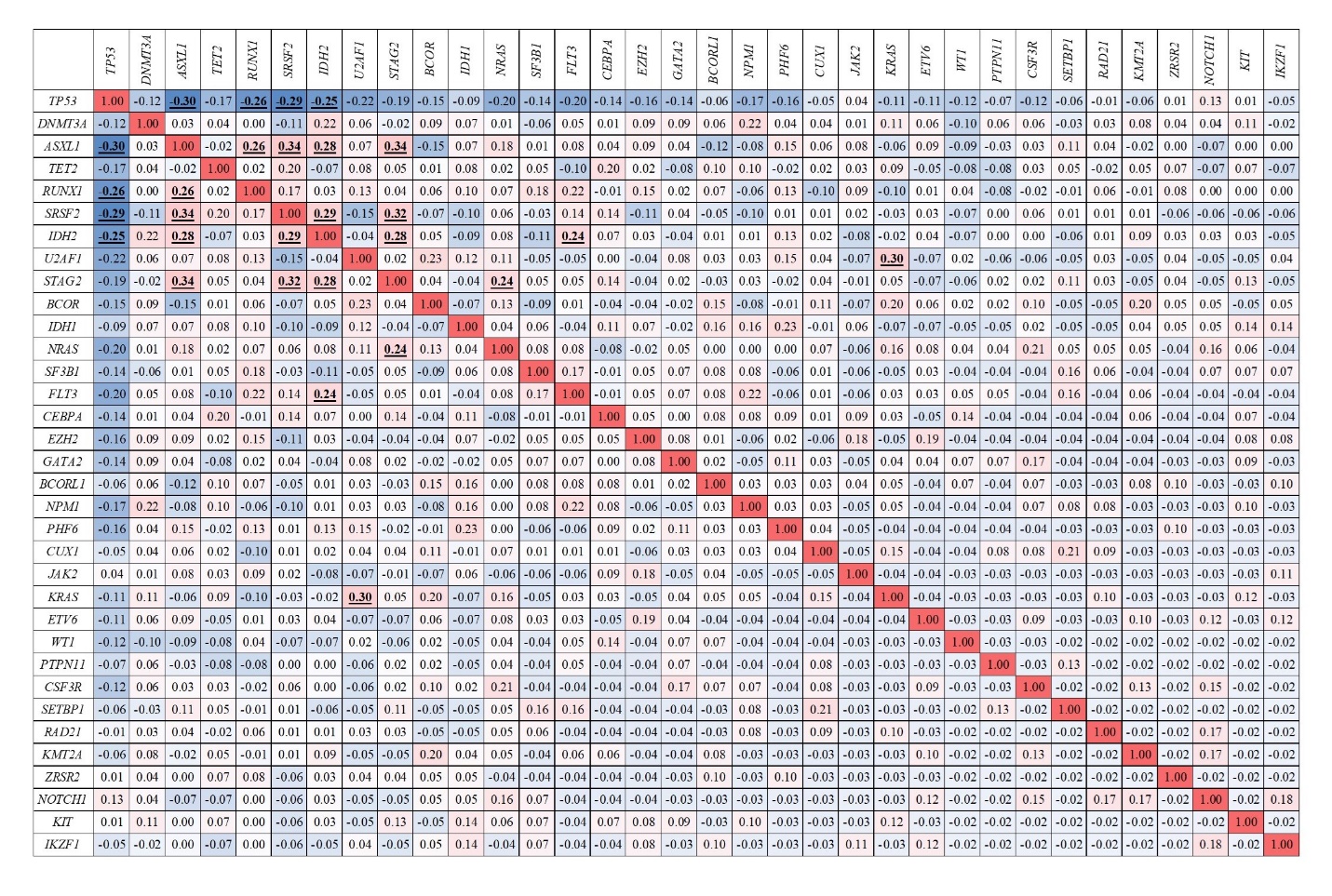


**Figure S3.** Kaplan-Meier estimates for OS and EFS of AML-MRC patients stratified by AML-MRC subtype.

**
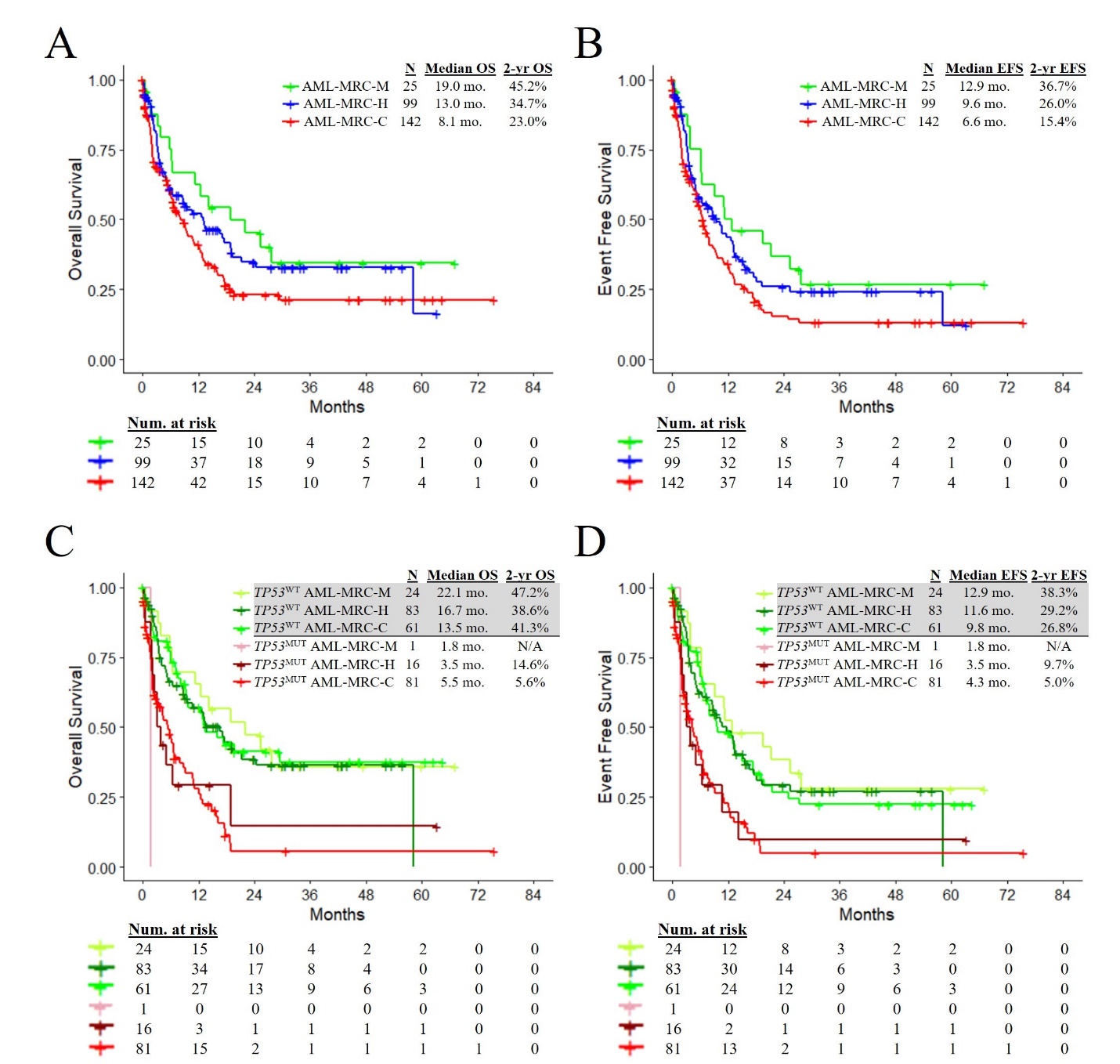
**

**Figure S4.** Kaplan-Meier estimates for OS and EFS of AML-MRC patients stratified by (A-B) *TP53* mutation status, (C-D) *IDH1* mutation status, (E-F) *NRAS* mutation status.


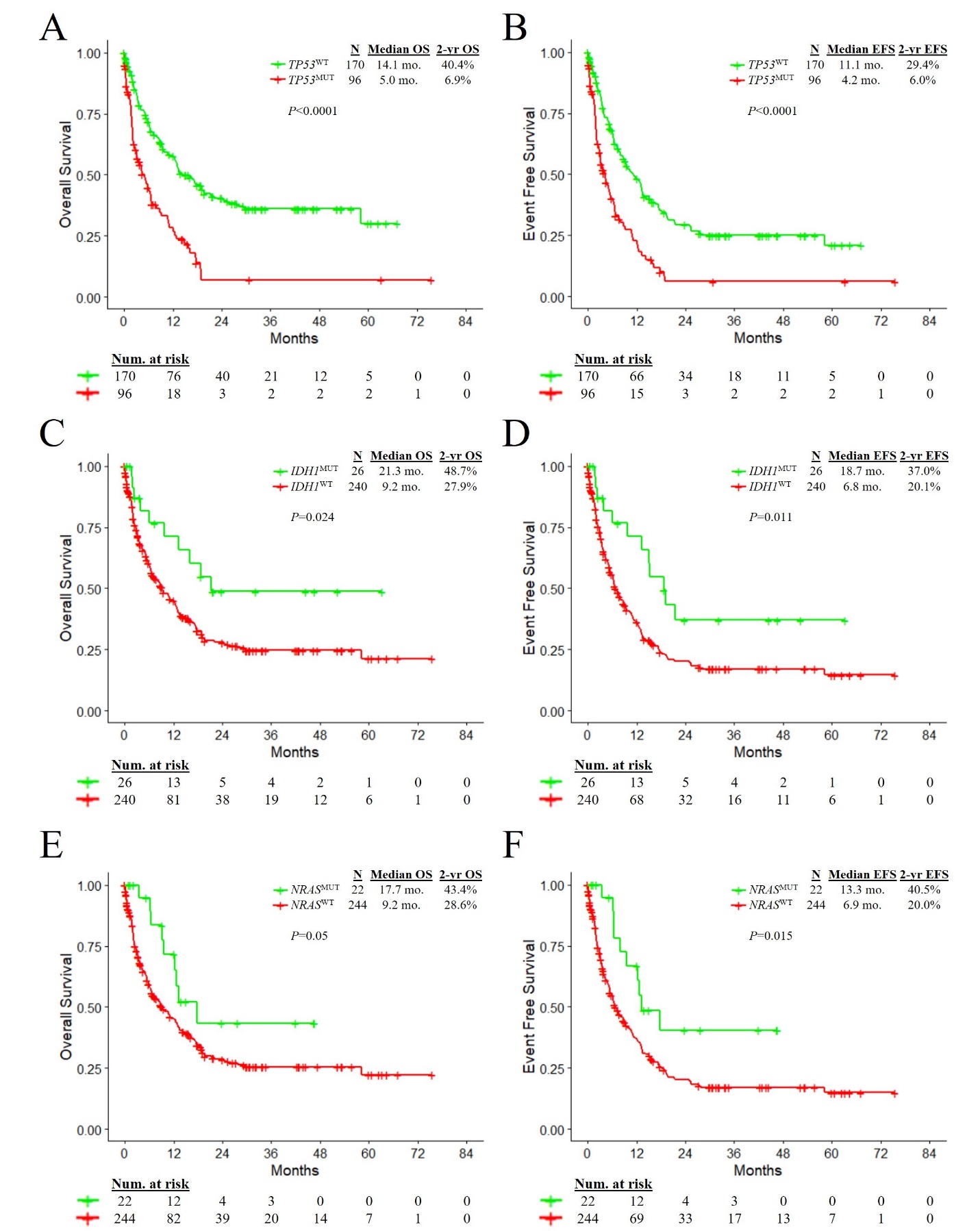


**Figure S5.** Kaplan-Meier estimates for OS and EFS of AML-MRC patients stratified by (A-B) *SF3B1* mutation status, and (C-D) *PHF6* mutation status.


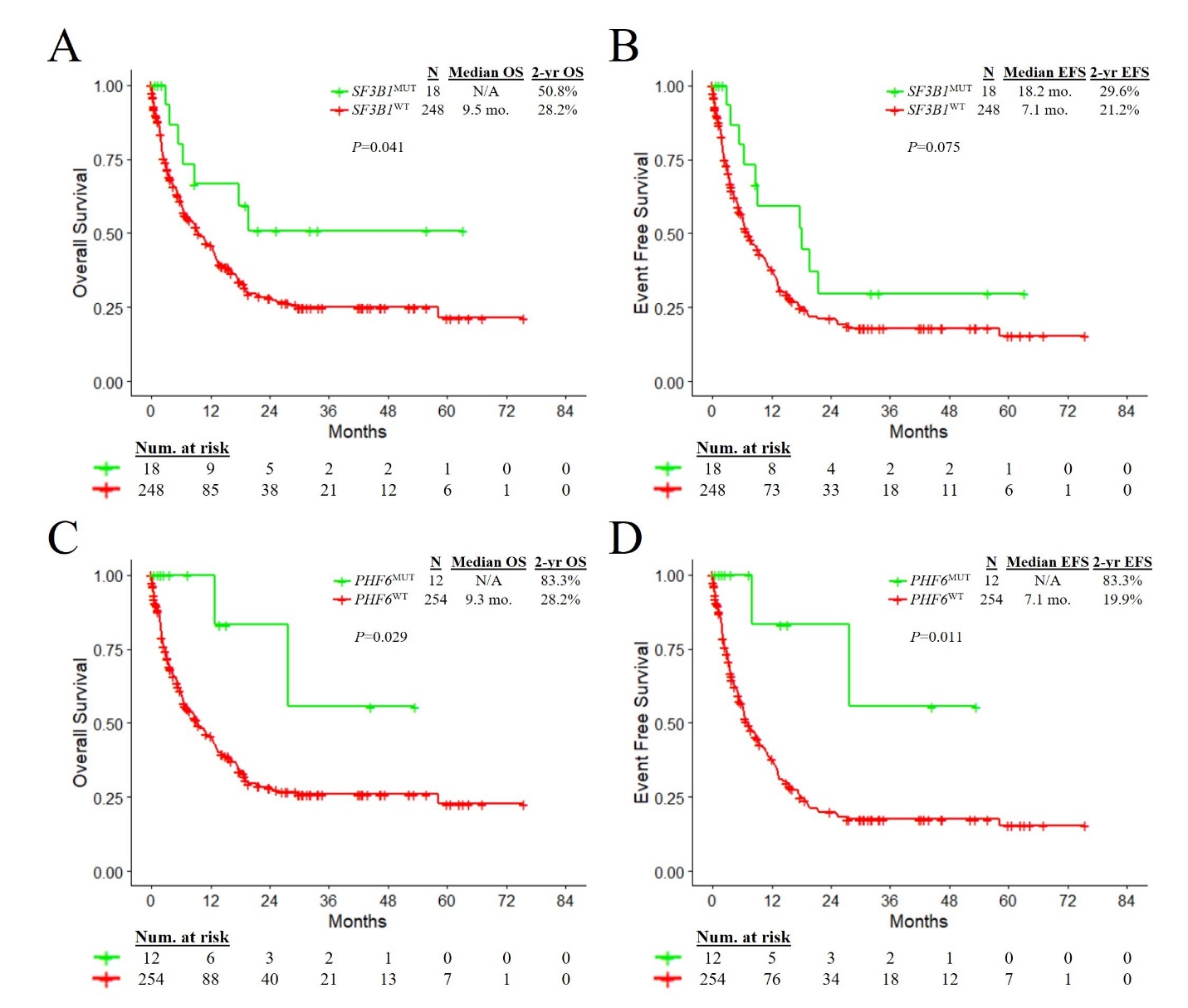


**Figure S6.** Kaplan-Meier estimates for OS and EFS of AML-MRC patients stratified by (A-B) presence or absence of cytogenetic loss of *TP53,* (C-D) number of *TP53* mutations, (E-F) both *TP53* mutation status and presence or absence of cytogenetic loss of *TP53*.


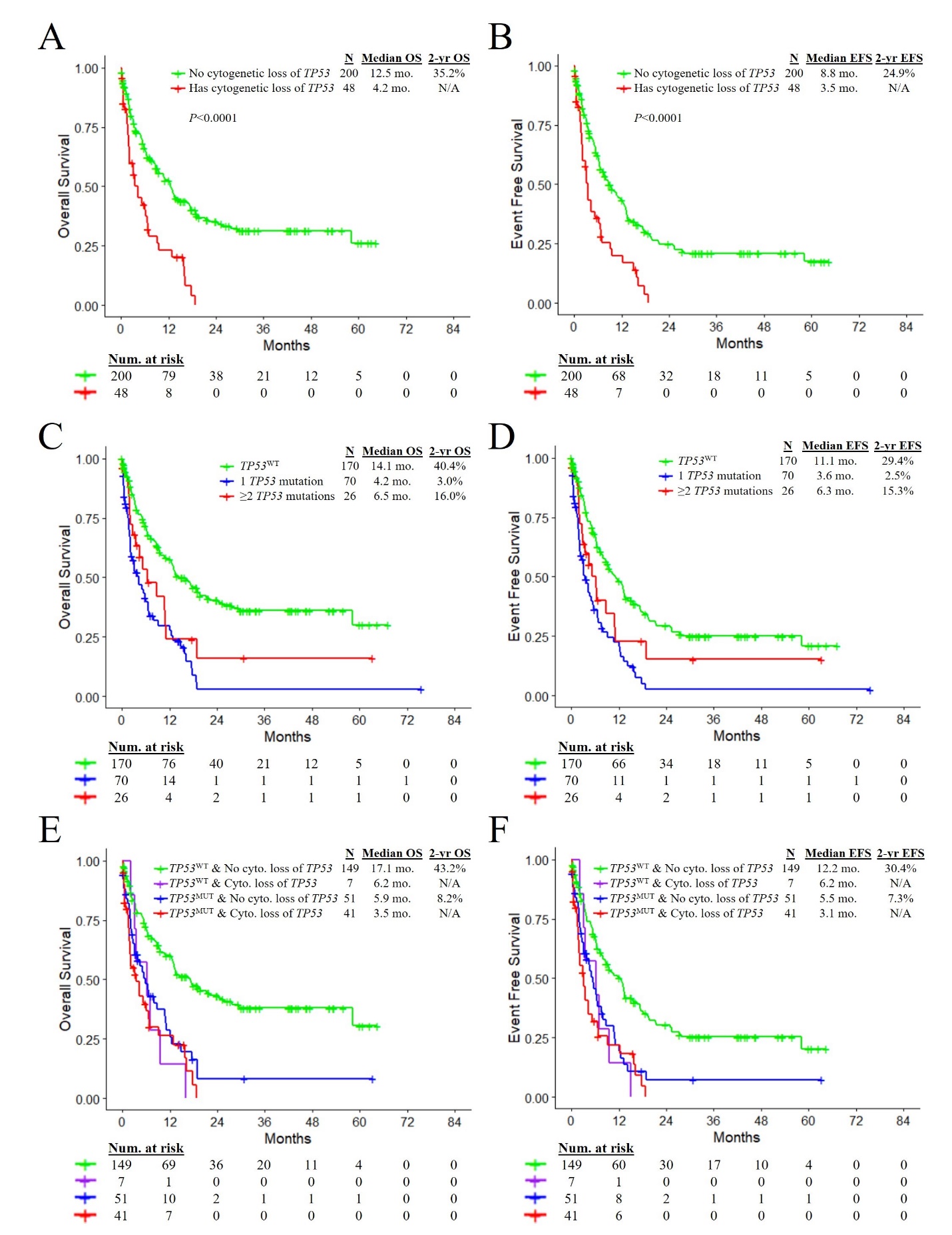


**Figure S7.** Schematic of clinical workflow to determine *TP53* allelic state based on *TP53* mutations and cytogenetic loss of chromosome 17p by karyotype assessment.


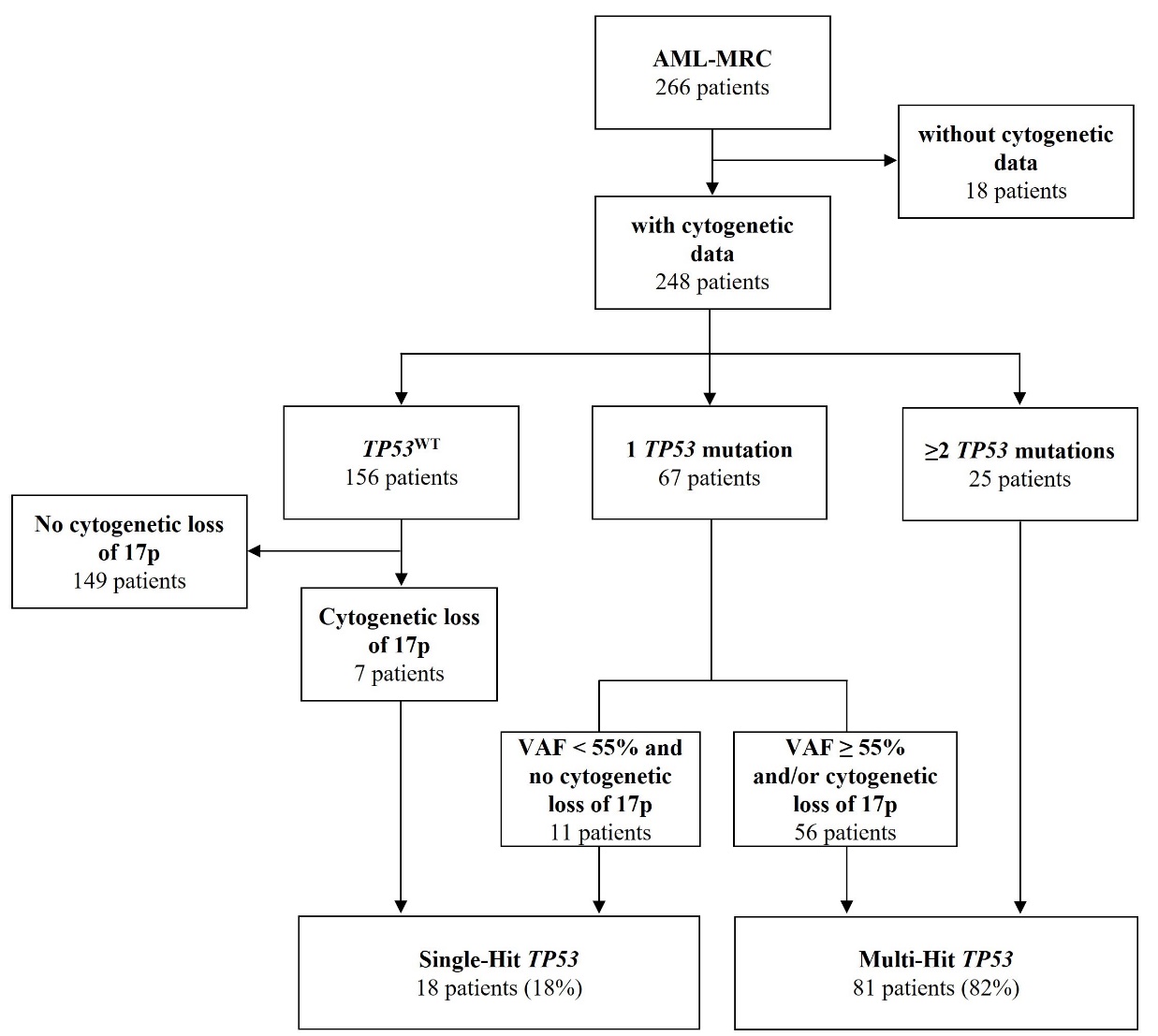


**Figure S8.** Kaplan-Meier estimates for OS and EFS of AML-MRC patients stratified by *TP53*^MUT^ variant allele frequency (VAF) threshold of 40%.


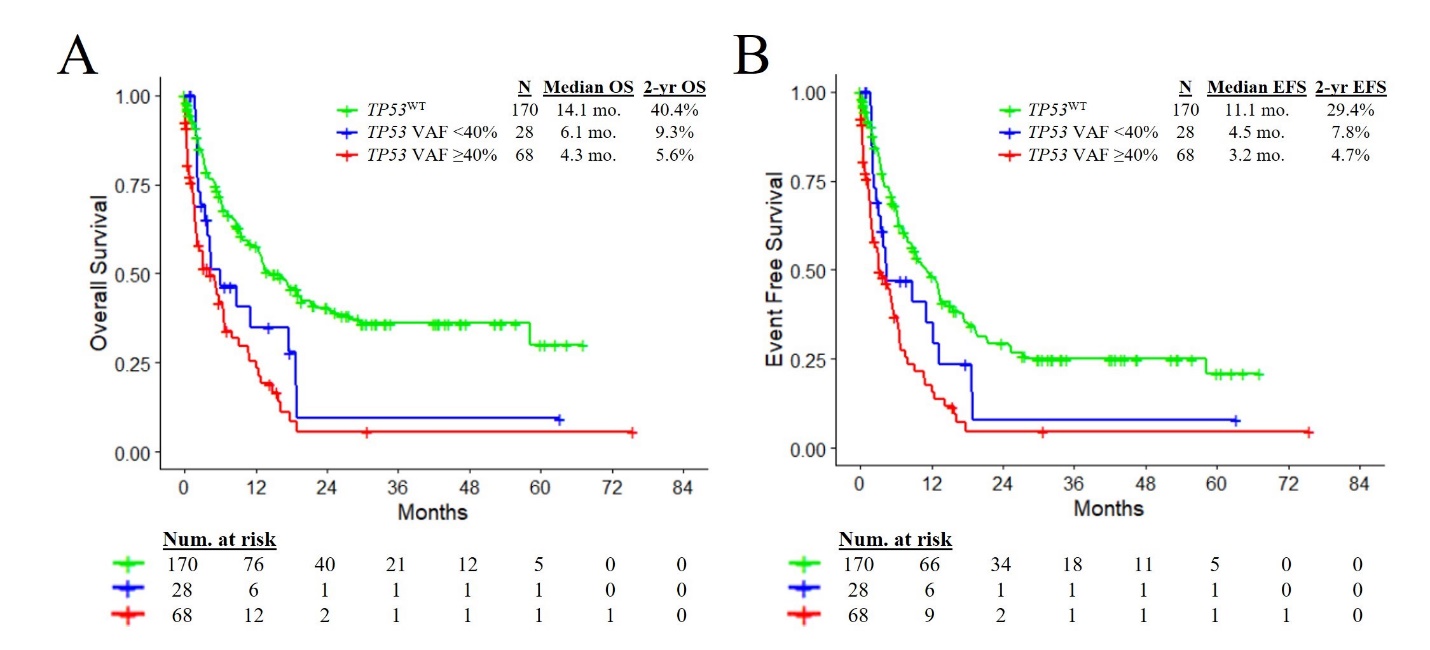


**Figure S9.** Kaplan-Meier estimates for OS and EFS of AML-MRC patients stratified by *TP53* mutation status and *DNMT3A* mutation status.


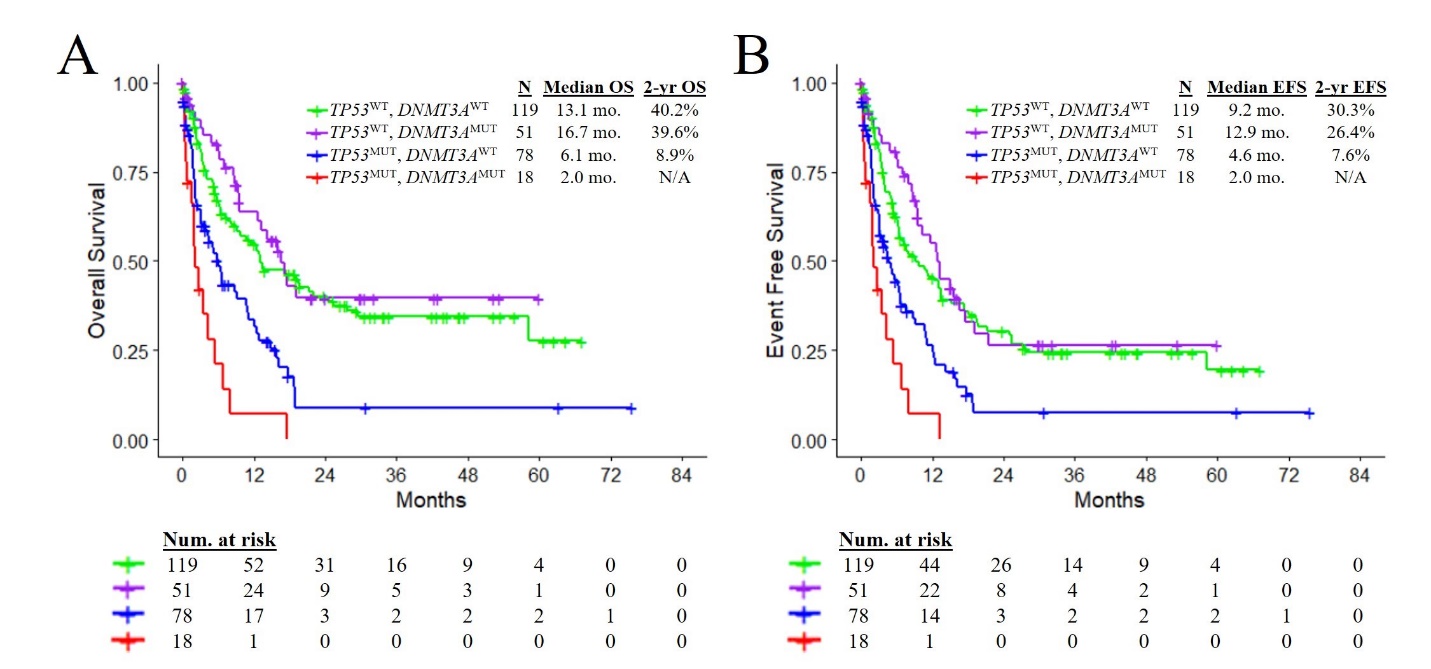


**Figure S10.** Kaplan-Meier estimates for OS and EFS of AML-MRC patients stratified by number of mutated genes using a threshold of (A-B) six mutated genes, (C-D) three mutated genes.


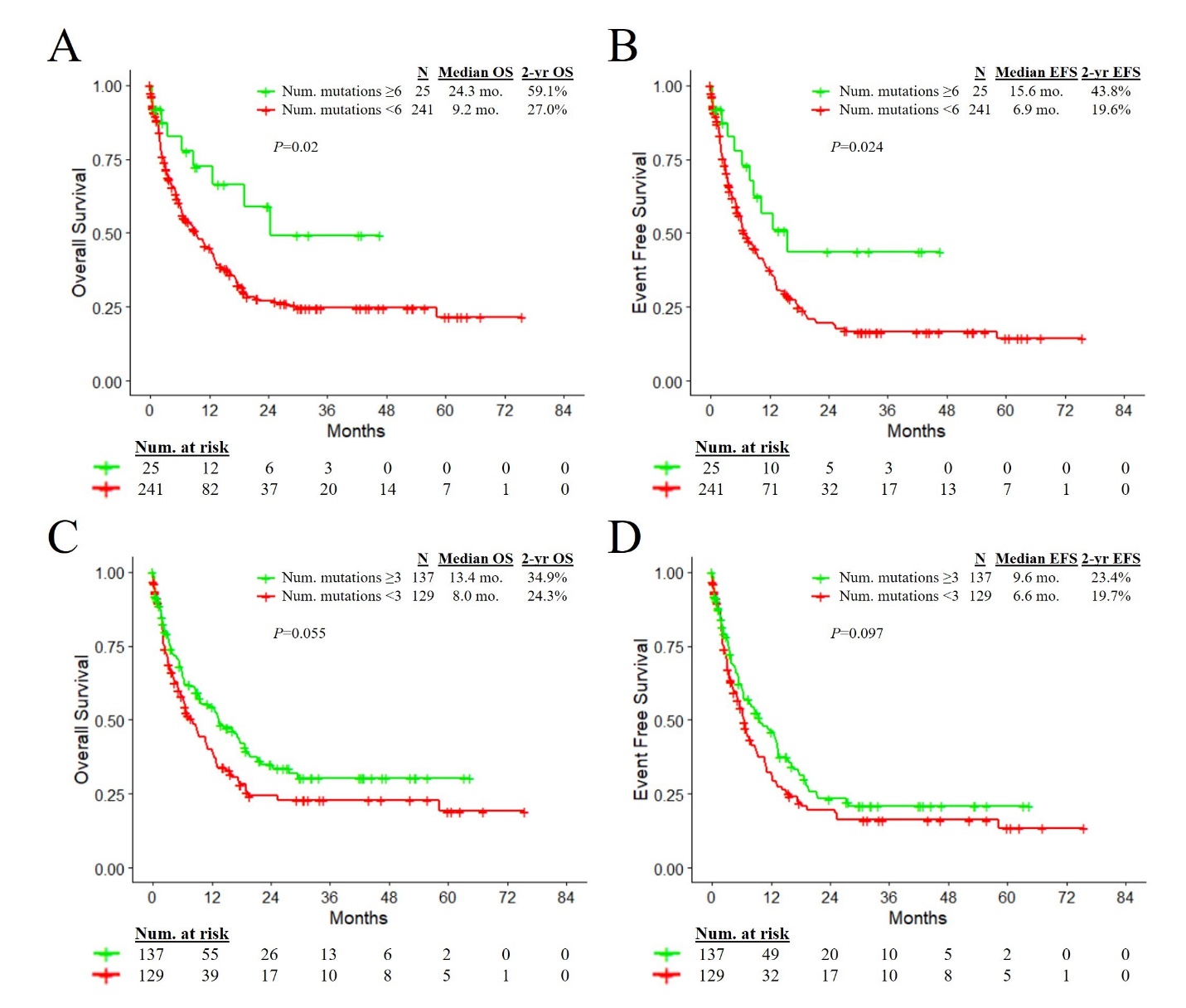


**Figure S11.** Kaplan-Meier estimates for (A) OS and (B) EFS of *TP53*^WT^ AML-MRC patients stratified by transplantation status. Kaplan-Meier estimates for (C) OS and (D) EFS of *TP53*^MUT^ AML-MRC patients stratified by transplantation status.


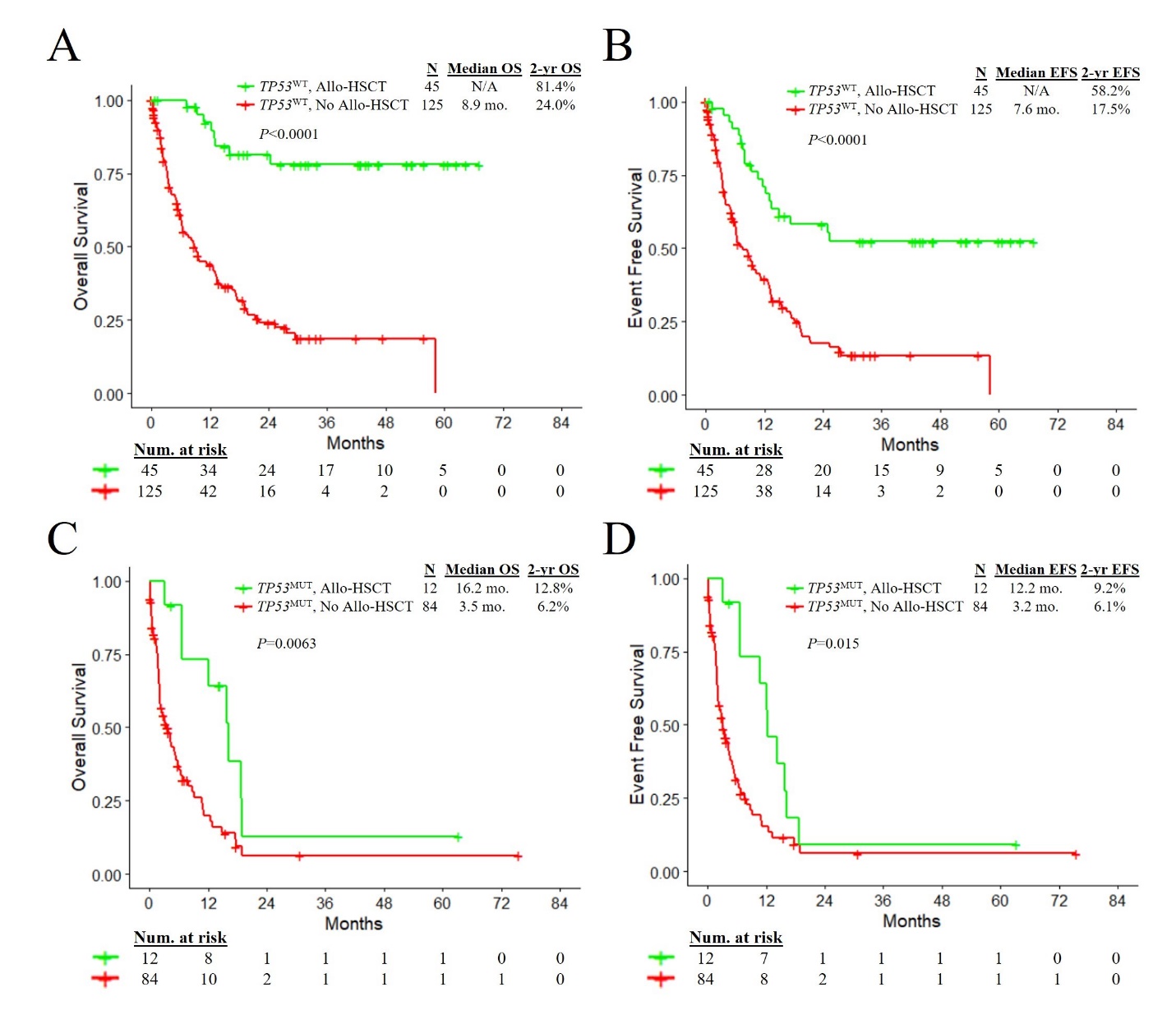


| **Table S1.** Gene panel for targeted sequencing | |
| --- | --- |
| **Complete coding region coverage (13/41)** | **Hotspot coverage (28/41)** |
| *BCOR* | *ASXL1* |
| *BCORL1* | *BRAF* |
| *CEBPA* | *CALR* |
| *CUX1* | *CBL* |
| *DNMT3A* | *CSF3R* |
| *ETV6* | *FBXW7* |
| *EZH2* | *FLT3* |
| *IKZF1* | *GATA2* |
| *PHF6* | *GNAS* |
| *RAD21* | *IDH1* |
| *RUNX1* | *IDH2* |
| *STAG2* | *JAK2* |
| *ZRSR2* | *KIT* |
|  | *KMT2A* |
|  | *KRAS* |
|  | *MPL* |
|  | *MYD88* |
|  | *NOTCH1* |
|  | *NPM1* |
|  | *NRAS* |
|  | *PTPN11* |
|  | *SETBP1* |
|  | *SF3B1* |
|  | *SRSF2* |
|  | *TET2* |
|  | *TP53* |
|  | *U2AF1* |
|  | *WT1* |

| **Table S2.** Exon coverage for hotspot genes | |
| --- | --- |
| **Gene** | **Exon Coverage** |
| *ASXL1* | 12 |
| *BRAF* | 15 |
| *CALR* | 9 |
| *CBL* | 8, 9 |
| *CSF3R* | 14-17 |
| *FBXW7* | 9-11 |
| *FLT3* | 14, 15, 20 |
| *GATA2* | 2-6 |
| *GNAS* | 8, 9 |
| *IDH1* | 4 |
| *IDH2* | 4 |
| *JAK2* | 12, 14 |
| *KIT* | 2, 8-11, 13, 17 |
| *KMT2A* | 5-8 |
| *KRAS* | 2,3 |
| *MPL* | 10 |
| *MYD88* | 3-5 |
| *NOTCH1* | 26-28, 34 |
| *NPM1* | 12 |
| *NRAS* | 2, 3 |
| *PTPN11* | 3, 13 |
| *SETBP1* | 4 |
| *SF3B1* | 13-16 |
| *SRSF2* | 1 |
| *TET2* | 3-11 |
| *TP53* | 2-11 |
| *U2AF1* | 2, 6 |
| *WT1* | 7, 9 |

| **Table S3.** List of mutations detected by NGS | | | | |
| --- | --- | --- | --- | --- |
| **Patient ID** | **Gene** | **Variant (cDNA)** | **Variant (AA)** | **VAF (%)** |
| *8751* | TP53 | c.645T>G | p.Ser215Arg | 48 |
| *8751* | TP53 | c.373A>C | p.Thr125Pro | 43 |
| *8751* | DNMT3A | c.1502A>G | p.Asn501Ser | 50 |
| *8751* | NOTCH1 | c.6509G>A | p.Ser2170Asn | 49 |
| *8751* | IKZF1 | c.476A>G | p.Asn159Ser | 9 |
| *105100* | SRSF2 | c.284C>A | p.Pro95His | 40 |
| *105100* | ASXL1 | c.2388G>A | p.Trp796* | 34 |
| *105100* | ASXL1 | c.2423C>A | p.Pro808His | 17 |
| *105100* | STAG2 | c.589_599delATGGATACAGT | p.Met197Hisfs*9 | 34 |
| *160804* | STAG2 | c.3097C>T | p.Arg1033* | 20 |
| *160804* | TET2 | c.4594C>T | p.Gln1532* | 14 |
| *160804* | CEBPA | c.199delT | p.Tyr67Thrfs*93 | 20 |
| *171493* | TP53 | c.618_619insTA | p.Asp207Ter | 81 |
| *171493* | ASXL1 | c.4099G>A | p.Val1367Ile | 53 |
| *171493* | DNMT3A | c.2644C>T | p.Arg882Cys | 43 |
| *205204* | U2AF1 | c.470A>C | p.Gln157Pro | 23 |
| *205204* | ASXL1 | c.2077C>T | p.Arg693* | 23 |
| *205204* | IDH1 | c.394C>T | p.Arg132Cys | 11 |
| *205204* | RUNX1 | c.1003_1015dupCAGTTCCCCGCGC | p.Leu339Profs*265 | 24 |
| *247449* | ASXL1 | c.1900_1922del | p.Glu635Argfs*15 | 47 |
| *247449* | DNMT3A | c.2645G>A | p.Arg882His | 39 |
| *247449* | IDH2 | c.419G>A | p.Arg140Gln | 35 |
| *276679* | TP53 | c.857A>C | p.Glu286Ala | 49 |
| *325425* | TP53 | c.329G>C | p.Arg110Pro | 40 |
| *325425* | DNMT3A | c.2478+1G>C | p.? | 37 |
| *325425* | TET2 | c.3478A>G | p.Ile1160Val | 48 |
| *451330* | BCORL1 | c.4258C>T | p.Arg1420* | 16 |
| *451330* | DNMT3A | c.2645G>A | p.Arg882His | 39 |
| *451330* | TET2 | c.521C>A | p.Pro174His | 51 |
| *451330* | IDH1 | c.394C>G | p.Arg132Gly | 38 |
| *451330* | RUNX1 | c.805+2dupT | p.? | 16 |
| *451330* | NPM1 | c.860_863dupTCTG | p.Trp288Cysfs*12 | 1 |
| *451330* | PHF6 | c.100delT | p.Ser34Leufs*47 | 21 |
| *554703* | TP53 | c.841G>A | p.Asp281Asn | 37 |
| *638373* | SRSF2 | c.284C>T | p.Pro95Leu | 36 |
| *638373* | TET2 | c.5650A>G | p.Thr1884Ala | 49 |
| *638373* | GATA2 | c.914T>G | p.Leu305Arg | 37 |
| *638373* | CSF3R | c.1919C>T | p.Thr640Ile | 54 |
| *638373* | CSF3R | c.1748G>A | p.Arg583His | 50 |
| *651203* | EZH2 | c.100C>T | p.Arg34Ter | 91 |
| *651203* | TET2 | c.3354delT | p.Leu1119TyrfsTer18 | 46 |
| *651203* | TET2 | c.4074C>G | p.Cys1358Trp | 55 |
| *651203* | CEBPA | c.584_589dupACCCGC | p.His195_Pro196dup | 44 |
| *804721* | TP53 | c.455dupC | p.Pro153Alafs*28 | 13 |
| *804721* | ASXL1 | c.1934dupG | p.Gly646Trpfs*12 | 19 |
| *804721* | DNMT3A | c.2645G>A | p.Arg882His | 42 |
| *804721* | IDH2 | c.419G>A | p.Arg140Gln | 36 |
| *804721* | CEBPA | c.56_58delinsCCT | p.Leu19_Gln20delinsPro* | 12 |
| *852417* | SF3B1 | c.2098A>G | p.Lys700Glu | 44 |
| *852417* | BCORL1 | c.1157delC | p.Pro386Glnfs*32 | 16 |
| *852417* | IKZF1 | c.476A>G | p.Asn159Ser | 45 |
| *904079* | SF3B1 | c.1876A>G | p.Asn626Asp | 47 |
| *904079* | ASXL1 | c.1934dupG | p.Gly646Trpfs*12 | 37 |
| *904079* | EZH2 | c.1449delT | p.Pro484Glnfs*30 | 45 |
| *904079* | RUNX1 | c.1003_1015dupCAGTTCCCCGCGC | p.Leu339Profs*265 | 34 |
| *904079* | ETV6 | c.212delA | p.Lys71Serfs*9 | 35 |
| *904079* | FLT3 | c.2503G>T | p.Asp835Tyr | 35 |
| *1069987* | TP53 | c.818G>A | p.Arg273His | 42 |
| *1069987* | TP53 | c.370dupT | p.Cys124Leufs*25 | 46 |
| *1069987* | BCORL1 | c.4435G>A | p.Gly1479Arg | 99 |
| *1087233* | TP53 | c.949C>T | p.Gln317* | 41 |
| *1087233* | TP53 | c.832C>T | p.Pro278Ser | 41 |
| *1138888* | DNMT3A | c.2644C>T | p.Arg882Cys | 16 |
| *1138888* | CSF3R | c.2503G>A | p.Glu835Lys | 50 |
| *1138888* | NPM1 | c.860_863dupTCTG | p.Trp288Cysfs*12 | 16 |
| *1140539* | TP53 | c.1031T>C | p.Leu344Pro | 90 |
| *1140539* | TET2 | c.5152G>T | p.Val1718Leu | 49 |
| *1235043* | TP53 | c.833C>G | p.Pro278Arg | 58 |
| *1925193* | DNMT3A | c.1627G>T | p.Gly543Cys | 36 |
| *1925193* | IDH2 | c.516_518delinsTGC | p.Arg172_His173delinsSerAla | 31 |
| *1972026* | SRSF2 | c.284C>A | p.Pro95His | 45 |
| *1972026* | ASXL1 | c.1896C>A | p.Cys632* | 42 |
| *1972026* | RUNX1 | c.331A>C | p.Thr111Pro | 84 |
| *1972026* | PHF6 | c.808delinsGGTT | p.Gln270_Asn365delinsGly | 31 |
| *2054561* | U2AF1 | c.101C>T | p.Ser34Phe | 31 |
| *2054561* | BCOR | c.1210C>T | p.Gln404* | 33 |
| *2054561* | RUNX1 | c.422_423insAAG | p.Ser141_Ala142insArg | 26 |
| *2054561* | WT1 | c.1109G>A | p.Arg370His | 47 |
| *2054561* | NRAS | c.35G>A | p.Gly12Asp | 12 |
| *2091426* | BCOR | c.4333C>T | p.Gln1445Ter | 14 |
| *2091426* | DNMT3A | c.1903C>T | p.Arg635Trp | 46 |
| *2091426* | IDH2 | c.419G>A | p.Arg140Gln | 17 |
| *2111556* | SRSF2 | c.284C>A | p.Pro95His | 42 |
| *2111556* | ASXL1 | c.1927_1928insA | p.Gly643Glufs*15 | 44 |
| *2111556* | STAG2 | c.3034C>T | p.Arg1012* | 74 |
| *2111556* | IDH2 | c.419G>A | p.Arg140Gln | 44 |
| *2111556* | NRAS | c.38G>T | p.Gly13Val | 30 |
| *2111556* | PHF6 | c.860G>C | p.Gly287Ala | 22 |
| *2124681* | SF3B1 | c.2098A>G | p.Lys700Glu | 32 |
| *2124681* | STAG2 | c.1535-2dupA | p.? | 35 |
| *2124681* | STAG2 | c.1535-3_1535-2insTA | p.? | 12 |
| *2124681* | DNMT3A | c.1687G>A | p.Val563Met | 26 |
| *2124681* | TET2 | c.5152G>T | p.Val1718Leu | 57 |
| *2124681* | IDH1 | c.394C>T | p.Arg132Cys | 31 |
| *2124681* | CEBPA | c.611delC | p.Pro204Argfs*114 | 22 |
| *2124681* | KIT | c.2446G>T | p.Asp816Tyr | 35 |
| *2124681* | NPM1 | c.863_864insCGTG | p.Trp288Cysfs*12 | 18 |
| *2124681* | NPM1 | c.863_864insCGAG | p.Trp288Cysfs*12 | 6 |
| *2132314* | ASXL1 | c.2959G>A | p.Gly987Arg | 50 |
| *2132314* | ASXL1 | c.2746A>T | p.Arg916* | 45 |
| *2132314* | KMT2A | c.2056A>G | p.Thr686Ala | 52 |
| *2132314* | TET2 | c.4681delT | p.Ser1561Leufs*10 | 44 |
| *2132314* | TET2 | c.5509delG | p.Ala1837Leufs*50 | 41 |
| *2134126* | SRSF2 | c.284C>G | p.Pro95Arg | 42 |
| *2134126* | ASXL1 | c.2485C>T | p.Gln829* | 41 |
| *2134126* | GATA2 | c.1193G>T | p.Arg398Leu | 43 |
| *2134126* | CSF3R | c.2087T>C | p.Met696Thr | 46 |
| *2164478* | SRSF2 | c.284C>A | p.Pro95His | 12 |
| *2164478* | SRSF2 | c.284C>G | p.Pro95Arg | 36 |
| *2164478* | ASXL1 | c.2387G>A | p.Trp796Ter | 11 |
| *2164478* | TET2 | c.3812dupG | p.Cys1271TrpfsTer29 | 11 |
| *2164478* | TET2 | c.987dupT | p.Glu330Ter | 28 |
| *2164478* | TET2 | c.5482C>T | p.Gln1828Ter | 49 |
| *2164478* | IDH2 | c.419G>A | p.Arg140Gln | 33 |
| *2164478* | CEBPA | c.542dupA | p.Tyr181Ter | 12 |
| *2179644* | TP53 | c.839G>C | p.Arg280Thr | 55 |
| *2179644* | DNMT3A | c.2716A>T | p.Lys906* | 37 |
| *2228105* | SRSF2 | c.284C>T | p.Pro95Leu | 44 |
| *2228105* | TET2 | c.3947delC | p.Pro1316Glnfs*47 | 45 |
| *2259944* | U2AF1 | c.101C>T | p.Ser34Phe | 31 |
| *2259944* | BCOR | c.4799_4800insA | p.Phe1600Leufs*8 | 95 |
| *2273861* | BCOR | c.1805dupC | p.Ala603Glyfs*8 | 31 |
| *2273861* | KMT2A | c.9685C>G | p.Leu3229Val | 49 |
| *2273861* | DNMT3A | c.2312G>A | p.Arg771Gln | 59 |
| *2273861* | IDH2 | c.515G>A | p.Arg172Lys | 38 |
| *2273861* | FLT3 | c.1769_1770ins24 | p.Tyr589_Phe590ins8 | 23 |
| *2293818* | ASXL1 | c.1771_1772insG | p.Tyr591Ter | 38 |
| *2293818* | STAG2 | c.2294_2295insG | p.Arg766GlufsTer19 | 33 |
| *2293818* | IDH2 | c.419G>A | p.Arg140Gln | 41 |
| *2314710* | DNMT3A | c.2645G>A | p.Arg882His | 40 |
| *2314710* | IDH1 | c.394C>A | p.Arg132Ser | 38 |
| *2314763* | U2AF1 | c.470A>C | p.Gln157Pro | 41 |
| *2314763* | ASXL1 | c.1934dupG | p.Gly646Trpfs*12 | 40 |
| *2314763* | EZH2 | c.965A>G | p.Asn322Ser | 49 |
| *2314763* | IDH1 | c.394C>T | p.Arg132Cys | 33 |
| *2314763* | CEBPA | c.377G>T | p.Gly126Val | 51 |
| *2314763* | GATA2 | c.1168_1170del | p.Lys390del | 7 |
| *2314763* | PHF6 | c.730-1_731del | p.? | 75 |
| *2314763* | PHF6 | c.737C>A | p.Ser246Tyr | 8 |
| *2320823* | ASXL1 | c.1934dupG | p.Gly646Trpfs*12 | 32 |
| *2320823* | FLT3 | c.2504A>T | p.Asp835Val | 42 |
| *2320823* | SETBP1 | c.2602G>A | p.Asp868Asn | 47 |
| *2359039* | TP53 | c.646G>A | p.Val216Met | 39 |
| *2359039* | TET2 | c.255T>G | p.Tyr85Ter | 25 |
| *2359039* | TET2 | c.3436C>T | p.Pro1146Ser | 37 |
| *2359039* | IDH1 | c.394C>T | p.Arg132Cys | 19 |
| *2419163* | SRSF2 | c.284C>A | p.Pro95His | 38 |
| *2419163* | BCORL1 | c.2336C>A | p.Pro779Gln | 100 |
| *2419163* | IDH2 | c.419G>A | p.Arg140Gln | 33 |
| *2419163* | FLT3 | c.1814_1815ins24 | p.Glu604_Phe605ins8 | 49 |
| *2596787* | SF3B1 | c.1997A>C | p.Lys666Thr | 49 |
| *2596787* | RUNX1 | c.421T>C | p.Ser141Pro | 45 |
| *2596787* | FLT3 | c.1782_1814dup | p.Arg595_Phe605dup | 39 |
| *2646416* | GATA2 | c.481C>G | p.Pro161Ala | 53 |
| *2646416* | WT1 | c.1138delinsGG | p.Arg380Glyfs*5 | 69 |
| *2651136* | PTPN11 | c.218C>T | p.Thr73Ile | 7 |
| *2669157* | TP53 | c.838A>T | p.Arg280* | 83 |
| *2671326* | U2AF1 | c.101C>T | p.Ser34Phe | 42 |
| *2671326* | NRAS | c.35G>A | p.Gly12Asp | 9 |
| *2671326* | KRAS | c.35G>A | p.Gly12Asp | 21 |
| *2684705* | TP53 | c.818G>A | p.Arg273His | 85 |
| *2684705* | SETBP1 | c.2602G>A | p.Asp868Asn | 13 |
| *2720515* | TP53 | c.743G>A | p.Arg248Gln | 47 |
| *2720515* | SF3B1 | c.1986C>A | p.His662Gln | 31 |
| *2720515* | RAD21 | c.1242T>G | p.Asp414Glu | 48 |
| *2720515* | NOTCH1 | c.6454G>C | p.Gly2152Arg | 47 |
| *2720515* | NOTCH1 | c.6397C>T | p.Pro2133Ser | 53 |
| *2720515* | NRAS | c.38G>A | p.Gly13Asp | 28 |
| *2720808* | CEBPA | c.798_799insTCTT | p.Gly267Serfs*55 | 78 |
| *2720808* | WT1 | c.1131_1138dupTCTTGTAC | p.Arg380Leufs*72 | 42 |
| *2743784* | U2AF1 | c.470A>C | p.Gln157Pro | 42 |
| *2743784* | SF3B1 | c.2233G>A | p.Ala745Thr | 16 |
| *2748334* | TP53 | c.659A>G | p.Tyr220Cys | 45 |
| *2772022* | DNMT3A | c.503delG | p.Gly168Alafs*57 | 31 |
| *2772022* | IDH2 | c.515G>A | p.Arg172Lys | 27 |
| *2772022* | RUNX1 | c.119_173del | p.Phe40Trpfs*14 | 35 |
| *2807397* | TP53 | c.722C>T | p.Ser241Phe | 10 |
| *2807397* | TP53 | c.394_395delinsTT | p.Lys132Leu | 13 |
| *2807397* | TP53 | c.102delC | p.Leu35CysfsTer9 | 8 |
| *2807397* | DNMT3A | c.2645G>A | p.Arg882His | 19 |
| *2836679* | SRSF2 | c.284C>A | p.Pro95His | 44 |
| *2836679* | IDH2 | c.419G>A | p.Arg140Gln | 51 |
| *2838357* | U2AF1 | c.101C>T | p.Ser34Phe | 38 |
| *2838357* | IDH1 | c.394C>T | p.Arg132Cys | 32 |
| *2838357* | IKZF1 | c.1298G>A | p.Arg433His | 59 |
| *2906754* | BCORL1 | c.2562dupC | p.Ser855Glnfs*32 | 86 |
| *2906754* | RUNX1 | c.467C>A | p.Ala156Glu | 74 |
| *2906754* | CEBPA | c.147_148insCCTG | p.Glu50Profs*59 | 38 |
| *2906754* | WT1 | c.1140dupG | p.Ser381Valfs*4 | 44 |
| *2906754* | WT1 | c.1107_1108insG | p.Arg370Alafs*15 | 44 |
| *2906754* | FLT3 | c.2522A>C | p.Asn841Thr | 42 |
| *2919949* | EZH2 | c.1037_1038insCCCGCTGA | p.Glu346AspfsTer6 | 36 |
| *2919949* | EZH2 | c.2050C>T | p.Arg684Cys | 16 |
| *2919949* | DNMT3A | c.1491delT | p.Cys497TrpfsTer154 | 38 |
| *2919949* | DNMT3A | c.2204A>G | p.Tyr735Cys | 38 |
| *2919949* | TET2 | c.2411T>C | p.Met804Thr | 48 |
| *2919949* | ETV6 | c.427C>T | p.Gln143Ter | 37 |
| *2923772* | TP53 | c.827C>G | p.Ala276Gly | 14 |
| *2923772* | TP53 | c.707A>G | p.Tyr236Cys | 13 |
| *2923772* | STAG2 | c.751G>C | p.Glu251Gln | 8 |
| *2985493* | SF3B1 | c.1986C>G | p.His662Gln | 46 |
| *2985493* | EZH2 | c.1882G>T | p.Gly628Cys | 50 |
| *2985493* | BCORL1 | c.5042delC | p.Pro1681Glnfs*20 | 21 |
| *2985493* | DNMT3A | c.2063G>T | p.Arg688Leu | 47 |
| *2985493* | DNMT3A | c.2098C>T | p.Pro700Ser | 51 |
| *2985493* | TET2 | c.4210C>T | p.Arg1404* | 12 |
| *2985493* | TET2 | c.1648C>T | p.Arg550* | 39 |
| *2985493* | RUNX1 | c.1059_1060insG | p.Thr354Aspfs*246 | 10 |
| *3037345* | WT1 | c.1390G>A | p.Asp464Asn | 22 |
| *3049133* | U2AF1 | c.101C>T | p.Ser34Phe | 40 |
| *3049133* | BCOR | c.4267C>T | p.Gln1423* | 82 |
| *3049133* | DNMT3A | c.2129G>C | p.Cys710Ser | 38 |
| *3049133* | DNMT3A | c.2644C>T | p.Arg882Cys | 40 |
| *3049133* | CUX1 | c.655C>T | p.Arg219* | 41 |
| *3049133* | CUX1 | c.4136G>A | p.Arg1379Gln | 55 |
| *3049133* | KRAS | c.179G>T | p.Gly60Val | 14 |
| *3054174* | ASXL1 | c.1934dupG | p.Gly646Trpfs*12 | 17 |
| *3054174* | EZH2 | c.2191T>C | p.Tyr731His | 23 |
| *3054174* | TET2 | c.521C>A | p.Pro174His | 49 |
| *3054174* | TET2 | c.4252delC | p.Leu1418Cysfs*30 | 18 |
| *3054174* | IDH1 | c.394C>T | p.Arg132Cys | 15 |
| *3054174* | RUNX1 | c.486G>T | p.Arg162Ser | 23 |
| *3059858* | TP53 | c.272G>A | p.Trp91* | 22 |
| *3059858* | TET2 | c.3259_3262delTCTT | p.Ser1087Glnfs*18 | 46 |
| *3059858* | TET2 | c.2280delT | p.Pro761Leufs*52 | 32 |
| *3078361* | TP53 | c.428T>A | p.Val143Glu | 46 |
| *3078361* | TP53 | c.772G>T | p.Glu258* | 40 |
| *3078361* | TET2 | c.3779A>G | p.Asn1260Ser | 48 |
| *3112923* | TP53 | c.646G>A | p.Val216Met | 79 |
| *3112923* | RAD21 | c.1576G>C | p.Glu526Gln | 23 |
| *3112923* | RUNX1 | c.1005G>T | p.Gln335His | 51 |
| *3130801* | TP53 | c.658T>G | p.Tyr220Asp | 19 |
| *3130801* | TP53 | c.310C>T | p.Gln104* | 15 |
| *3130801* | DNMT3A | c.1077C>A | p.Tyr359* | 18 |
| *3196462* | TP53 | c.659A>G | p.Tyr220Cys | 12 |
| *3200065* | U2AF1 | c.101C>T | p.Ser34Phe | 42 |
| *3200065* | BCOR | c.3649dupC | p.Arg1217Profs*28 | 10 |
| *3200065* | RUNX1 | c.254_276dup | p.Asp93Thrfs*37 | 4 |
| *3200065* | NRAS | c.183A>T | p.Gln61His | 38 |
| *3200065* | FLT3 | c.1793_1864dup | p.Phe621_Gly622ins54 | 14 |
| *3200065* | CBL | c.1099C>A | p.Gln367Lys | 5 |
| *3201721* | SRSF2 | c.282_284delinsGCA | p.Pro95His | 47 |
| *3201721* | TET2 | c.3799G>T | p.Glu1267* | 46 |
| *3201721* | TET2 | c.5238_5242dupTCCAA | p.Asn1748Ilefs*17 | 38 |
| *3279805* | TP53 | c.659A>G | p.Tyr220Cys | 41 |
| *3279805* | TP53 | c.818G>A | p.Arg273His | 42 |
| *3282425* | EZH2 | c.2125G>A | p.Gly709Ser | 11 |
| *3282425* | JAK2 | c.1849G>T | p.Val617Phe | 18 |
| *3300288* | U2AF1 | c.101C>T | p.Ser34Phe | 48 |
| *3300288* | BCOR | c.4173+1G>A | p.? | 46 |
| *3300288* | STAG2 | c.2230C>T | p.Gln744* | 43 |
| *3300288* | STAG2 | c.1553A>C | p.Glu518Ala | 48 |
| *3300288* | DNMT3A | c.802dupG | p.Asp268Glyfs*13 | 45 |
| *3300288* | TET2 | c.769A>G | p.Thr257Ala | 50 |
| *3300288* | TET2 | c.775G>T | p.Glu259* | 47 |
| *3300288* | KRAS | c.182A>G | p.Gln61Arg | 33 |
| *3317938* | TP53 | c.843C>A | p.Asp281Glu | 44 |
| *3361496* | TP53 | c.818G>A | p.Arg273His | 61 |
| *3361496* | DNMT3A | c.89A>C | p.Glu30Ala | 48 |
| *3362313* | ASXL1 | c.1934dupG | p.Gly646Trpfs*12 | 34 |
| *3362313* | RUNX1 | c.287A>T | p.Asn96Ile | 10 |
| *3400645* | TP53 | c.659A>G | p.Tyr220Cys | 36 |
| *3402102* | U2AF1 | c.101C>T | p.Ser34Phe | 36 |
| *3402102* | ASXL1 | c.1773C>A | p.Tyr591Ter | 38 |
| *3402102* | DNMT3A | c.1299_1302delAGAA | p.Glu434CysfsTer216 | 40 |
| *3459100* | BCOR | c.3117dupA | p.Asp1040Argfs*39 | 77 |
| *3459100* | ETV6 | c.262_315dup | p.Glu88_Arg105dup | 47 |
| *3459100* | IKZF1 | c.476A>G | p.Asn159Ser | 21 |
| *3570781* | ASXL1 | c.1900_1922delAGAGAGGCGGCCACCACTGCCAT | p.Glu635Argfs*15 | 39 |
| *3578553* | TP53 | c.398T>A | p.Met133Lys | 87 |
| *3596788* | TP53 | c.818G>A | p.Arg273His | 52 |
| *3596788* | ASXL1 | c.2530dupA | p.Thr844Asnfs*7 | 31 |
| *3596788* | STAG2 | c.2984T>A | p.Leu995* | 6 |
| *3596788* | JAK2 | c.1849G>T | p.Val617Phe | 5 |
| *3638065* | TP53 | c.818G>A | p.Arg273His | 29 |
| *3638065* | TP53 | c.659A>G | p.Tyr220Cys | 37 |
| *3638065* | BCORL1 | c.3636delT | p.Phe1213SerfsTer9 | 59 |
| *3638065* | GATA2 | c.856G>A | p.Ala286Thr | 40 |
| *3722941* | SRSF2 | c.284C>A | p.Pro95His | 43 |
| *3722941* | TET2 | c.2820_2823delCCCT | p.Pro942ArgfsTer10 | 47 |
| *3722941* | CEBPA | c.68dupC | p.His24AlafsTer84 | 17 |
| *3726199* | U2AF1 | c.101C>T | p.Ser34Phe | 47 |
| *3726199* | TET2 | c.5972T>G | p.Phe1991Cys | 52 |
| *3726199* | IDH1 | c.394C>T | p.Arg132Cys | 46 |
| *3731125* | SRSF2 | c.284C>G | p.Pro95Arg | 38 |
| *3731125* | KMT2A | c.10318A>G | p.Ile3440Val | 50 |
| *3731125* | TET2 | c.3955-2A>G | p.? | 42 |
| *3731125* | RUNX1 | c.592G>A | p.Asp198Asn | 52 |
| *3736150* | TP53 | c.844C>T | p.Arg282Trp | 51 |
| *3736150* | TP53 | c.818G>A | p.Arg273His | 43 |
| *3774063* | TP53 | c.586C>T | p.Arg196* | 80 |
| *3783601* | ZRSR2 | c.205C>T | p.Gln69* | 79 |
| *3783601* | IDH1 | c.394C>T | p.Arg132Cys | 45 |
| *3783601* | RUNX1 | c.497G>A | p.Arg166Gln | 55 |
| *3783601* | PHF6 | c.983dupA | p.Asn328Lysfs*3 | 79 |
| *3827006* | SRSF2 | c.284C>A | p.Pro95His | 48 |
| *3827006* | DNMT3A | c.2644C>T | p.Arg882Cys | 37 |
| *3827006* | IDH2 | c.419G>A | p.Arg140Gln | 28 |
| *3827006* | ETV6 | c.1004delT | p.Ile335Lysfs*36 | 6 |
| *3827006* | ETV6 | c.1106G>A | p.Arg369Gln | 6 |
| *3838465* | TP53 | c.730G>T | p.Gly244Cys | 60 |
| *3838465* | TP53 | c.701A>G | p.Tyr234Cys | 24 |
| *3838465* | ZRSR2 | c.18G>C | p.Lys6Asn | 100 |
| *3882761* | SF3B1 | c.2098A>G | p.Lys700Glu | 47 |
| *3882761* | RUNX1 | c.496C>T | p.Arg166* | 33 |
| *3897799* | TP53 | c.498dupA | p.Gln167ThrfsTer14 | 31 |
| *3897799* | IDH1 | c.394C>T | p.Arg132Cys | 26 |
| *3897799* | NOTCH1 | c.6853G>A | p.Val2285Ile | 50 |
| *3908880* | TP53 | c.875_893del | p.Lys292Serfs*47 | 59 |
| *3908880* | TP53 | c.358A>G | p.Lys120Glu | 42 |
| *3914841* | TP53 | c.782+1G>A | p.? | 82 |
| *3918244* | DNMT3A | c.2096G>A | p.Gly699Asp | 51 |
| *3984192* | TP53 | c.858delA | p.Glu287Argfs*58 | 61 |
| *3984192* | ASXL1 | c.1934dupG | p.Gly646Trpfs*12 | 21 |
| *3984192* | DNMT3A | c.2204A>G | p.Tyr735Cys | 33 |
| *4087781* | TP53 | c.733G>T | p.Gly245Cys | 66 |
| *4087781* | RUNX1 | c.120C>G | p.Phe40Leu | 59 |
| *4087781* | JAK2 | c.1849G>T | p.Val617Phe | 92 |
| *4094823* | SRSF2 | c.284C>T | p.Pro95Leu | 49 |
| *4094823* | DNMT3A | c.2645G>A | p.Arg882His | 47 |
| *4094823* | TET2 | c.3594+1_3594+2insT | p.? | 35 |
| *4094823* | TET2 | c.4019T>C | p.Leu1340Pro | 54 |
| *4097419* | TP53 | c.217delG | p.V73Wfs*50 | 60 |
| *4097419* | DNMT3A | c.2726T>C | p.F909S | 43 |
| *4122222* | TP53 | c.817C>T | p.Arg273Cys | 62 |
| *4164554* | TET2 | c.3968A>T | p.Glu1323Val | 10 |
| *4168198* | SRSF2 | c.284C>A | p.Pro95His | 44 |
| *4168198* | STAG2 | c.753_754insTTAC | p.Arg252LeufsTer4 | 76 |
| *4168198* | RUNX1 | c.530T>G | p.Ile177Ser | 37 |
| *4173094* | BCOR | c.4180G>C | p.Val1394Leu | 79 |
| *4173094* | PHF6 | c.977G>A | p.Cys326Tyr | 83 |
| *4176060* | DNMT3A | c.2309C>T | p.Ser770Leu | 35 |
| *4176060* | GATA2 | c.1085G>A | p.Arg362Gln | 15 |
| *4176797* | TP53 | c.772G>C | p.Glu258Gln | 67 |
| *4176797* | ASXL1 | c.2957A>G | p.Asn986Ser | 51 |
| *4181199* | U2AF1 | c.470A>C | p.Gln157Pro | 40 |
| *4181199* | BCOR | c.7T>A | p.Ser3Thr | 12 |
| *4181199* | TET2 | c.100C>T | p.Leu34Phe | 53 |
| *4181199* | RUNX1 | c.964_965delTC | p.Ser322AsnfsTer277 | 43 |
| *4181199* | RUNX1 | c.156_157insCTCT | p.Ser53LeufsTer2 | 25 |
| *4182031* | SF3B1 | c.1998G>C | p.Lys666Asn | 47 |
| *4182031* | RUNX1 | c.610C>T | p.Arg204Ter | 47 |
| *4182031* | GATA2 | c.974_975insACT | p.Met325delinsIleLeu | 43 |
| *4182031* | FLT3 | c.2503G>T | p.Asp835Tyr | 40 |
| *4183131* | TP53 | c.404G>T | p.Cys135Phe | 63 |
| *4183131* | TET2 | c.992T>G | p.Ile331Arg | 44 |
| *4183131* | KRAS | c.38G>A | p.Gly13Asp | 19 |
| *4183131* | KIT | c.251C>T | p.Thr84Met | 51 |
| *4187126* | SRSF2 | c.284C>A | p.Pro95His | 52 |
| *4187126* | SF3B1 | c.2242A>G | p.Lys748Glu | 49 |
| *4187126* | ASXL1 | c.2423delC | FS | 37 |
| *4187126* | STAG2 | c.2730delA | FS | 11 |
| *4187126* | TET2 | c.1630C>T | p.Arg544X | 40 |
| *4187126* | TET2 | c.3443A>C | p.Tyr1148Ser | 46 |
| *4187126* | CUX1 | c.2164C>T | p.Gln722X | 83 |
| *4187126* | NRAS | c.37G>C | p.Gly13Arg | 37 |
| *4187126* | SETBP1 | c.2612T>C | p.Ile871Thr | 48 |
| *4190732* | TP53 | c.524G>A | p.Arg175His | 54 |
| *4190732* | U2AF1 | c.470A>C | p.Gln157Pro | 35 |
| *4190732* | ZRSR2 | c.283G>A | p.Ala95Thr | 100 |
| *4190732* | DNMT3A | c.2193_2195delCTT | p.Phe732del | 33 |
| *4190732* | RUNX1 | c.1005G>T | p.Gln335His | 50 |
| *4194485* | SRSF2 | c.284C>A | p.Pro95His | 48 |
| *4194485* | DNMT3A | c.2678G>C | p.Trp893Ser | 47 |
| *4194485* | TET2 | c.5396delA | p.Lys1799Argfs*21 | 92 |
| *4194485* | RUNX1 | c.509-2A>C | p.? | 47 |
| *4194485* | CEBPA | c.540_541dupTT | p.Tyr181Phefs*138 | 6 |
| *4194485* | JAK2 | c.1849G>T | p.Val617Phe | 45 |
| *4198478* | FLT3 | c.1765T>C | p.Tyr589His | 17 |
| *4198478* | NPM1 | c.863_864insTCGG | p.Trp288CysfsTer12 | 7 |
| *4199650* | SRSF2 | c.284C>A | p.Pro95His | 12 |
| *4199650* | IDH2 | c.419G>A | p.Arg140Gln | 12 |
| *4211654* | BCOR | c.3830C>A | p.Pro1277His | 11 |
| *4211654* | BCOR | c.2296G>T | p.Glu766Ter | 72 |
| *4211654* | RUNX1 | c.1065C>G | p.Tyr355Ter | 63 |
| *4220447* | ASXL1 | c.2387G>A | p.Trp796Ter | 45 |
| *4220447* | DNMT3A | c.2645G>A | p.Arg882His | 48 |
| *4220447* | GATA2 | c.1075T>G | p.Leu359Val | 46 |
| *4220447* | ETV6 | c.872G>A | p.Arg291Lys | 48 |
| *4220447* | NRAS | c.182A>T | p.Gln61Leu | 51 |
| *4223343* | TP53 | c.524G>A | p.Arg175His | 28 |
| *4223343* | TP53 | c.716A>G | p.Asn239Ser | 29 |
| *4223343* | U2AF1 | c.470A>C | p.Gln157Pro | 29 |
| *4223906* | SRSF2 | c.284C>A | p.Pro95His | 49 |
| *4223906* | STAG2 | c.759_760insA | p.Met255fs | 69 |
| *4223906* | IDH2 | c.419G>A | p.Arg140Gln | 39 |
| *4223906* | CEBPA | c.189_190dupCA | p.Ile64fs | 26 |
| *4223906* | CEBPA | c.198_201dupCTAC | p.Ile68fs | 18 |
| *4232505* | DNMT3A | c.2645G>A | p.Arg882His | 34 |
| *4235048* | SRSF2 | c.284C>T | p.Pro95Leu | 27 |
| *4235048* | ASXL1 | c.2323delT | p.Leu775Ter | 22 |
| *4235048* | TET2 | c.3860T>C | p.Phe1287Ser | 9 |
| *4235048* | TET2 | c.232delT | p.Phe78LeufsTer17 | 26 |
| *4235048* | RUNX1 | c.1163C>A | p.Ser388Ter | 24 |
| *4235048* | JAK2 | c.1849G>T | p.Val617Phe | 13 |
| *4237034* | STAG2 | c.958_959insT | p.Ser320MetfsTer2 | 23 |
| *4241837* | SRSF2 | c.284C>A | p.Pro95His | 25 |
| *4241837* | ASXL1 | c.1772dup | p.Tyr591Ter | 20 |
| *4241837* | BCOR | c.4247_4248insG | p.Pro1418AlafsTer43 | 32 |
| *4241837* | STAG2 | c.3644_3650delCAGAAGG | p.Ser1215Ter | 40 |
| *4241837* | RUNX1 | c.497G>A | p.Arg166Gln | 16 |
| *4242445* | TP53 | c.584T>C | p.Ile195Thr | 82 |
| *4245769* | TP53 | c.473G>A | p.Arg158His | 27 |
| *4245769* | TP53 | c.581T>G | p.Leu194Arg | 41 |
| *4248101* | ASXL1 | c.2423delC | p.Pro808LeufsTer10 | 44 |
| *4248101* | ASXL1 | c.1898A>G | p.His633Arg | 55 |
| *4248101* | EZH2 | c.2041G>A | p.Asp681Asn | 89 |
| *4248101* | DNMT3A | c.2645G>A | p.Arg882His | 52 |
| *4248101* | IDH2 | c.419G>A | p.Arg140Gln | 49 |
| *4252623* | U2AF1 | c.101C>A | p.Ser34Tyr | 25 |
| *4252623* | TET2 | c.744C>A | p.His248Gln | 49 |
| *4252623* | TET2 | c.1285G>A | p.Gly429Arg | 48 |
| *4252623* | TET2 | c.5152G>T | p.Val1718Leu | 52 |
| *4253243* | TP53 | c.880G>T | p.Glu294Ter | 43 |
| *4263981* | BCOR | c.1981C>G | p.Pro661Ala | 99 |
| *4263981* | CEBPA | c.107delG | p.Gly36Alafs*124 | 71 |
| *4263981* | CUX1 | c.2363C>T | p.Ala788Val | 54 |
| *4266734* | TP53 | c.743G>A | p.Arg248Gln | 79 |
| *4268253* | SRSF2 | c.284C>A | p.Pro95His | 49 |
| *4268253* | ASXL1 | c.2077C>T | p.Arg693* | 41 |
| *4268253* | STAG2 | c.1840C>T | p.Arg614* | 74 |
| *4268253* | TET2 | c.3646C>G | p.Arg1216Gly | 41 |
| *4268253* | RUNX1 | c.316T>C | p.Trp106Arg | 41 |
| *4268253* | CEBPA | c.68dupC | p.His24Alafs*84 | 13 |
| *4269023* | TP53 | c.743G>A | p.Arg248Gln | 61 |
| *4269023* | TET2 | c.3595-1G>A | p.? | 30 |
| *4269023* | TET2 | c.5885C>T | p.Pro1962Leu | 49 |
| *4269023* | CUX1 | c.1336C>A | p.Pro446Thr | 51 |
| *4273266* | RAD21 | c.790A>G | p.Met264Val | 47 |
| *4273266* | CUX1 | c.295G>A | p.Val99Ile | 64 |
| *4273266* | KRAS | c.38G>A | p.Gly13Asp | 7 |
| *4276576* | EZH2 | c.1410+1_1410+2insACAGG | p.? | 58 |
| *4281883* | SRSF2 | c.284C>T | p.Pro95Leu | 27 |
| *4281883* | RUNX1 | c.497G>A | p.Arg166Gln | 42 |
| *4282823* | U2AF1 | c.470A>G | p.Gln157Arg | 22 |
| *4282823* | DNMT3A | c.2645G>A | p.Arg882His | 25 |
| *4282823* | TET2 | c.5103G>A | p.Met1701Ile | 48 |
| *4282823* | CEBPA | c.985delG | p.Glu329Asnfs*3 | 21 |
| *4282823* | KRAS | c.35G>A | p.Gly12Asp | 24 |
| *4282823* | NPM1 | c.860_863dupTCTG | p.Trp288Cysfs*12 | 18 |
| *4286939* | ASXL1 | c.1934dupG | p.Gly646Trpfs*12 | 37 |
| *4286939* | DNMT3A | c.2644C>T | p.Arg882Cys | 50 |
| *4286939* | IDH2 | c.419G>A | p.Arg140Gln | 50 |
| *4286939* | RUNX1 | c.505dupA | p.Arg169Lysfs*44 | 44 |
| *4287060* | SRSF2 | c.284C>A | p.Pro95His | 50 |
| *4287060* | ASXL1 | c.1900_1922delAGAGAGGCGGCCACCACTGCCAT | p.Glu635Argfs*15 | 40 |
| *4287060* | IDH2 | c.419G>A | p.Arg140Gln | 49 |
| *4287060* | RUNX1 | c.610C>T | p.Arg204* | 49 |
| *4287060* | FLT3 | c.1833_1834ins48 | p.Glu611_Phe612ins16 | 6 |
| *4291585* | U2AF1 | c.470A>C | p.Gln157Pro | 44 |
| *4291585* | ASXL1 | c.1900_1922delAGAGAGGCGGCCACCACTGCCAT | p.Glu635Argfs*15 | 58 |
| *4291585* | IDH2 | c.419G>A | p.Arg140Gln | 42 |
| *4291585* | RUNX1 | c.1301dupA | p.Asn434Lysfs*166 | 33 |
| *4291585* | PHF6 | c.375-1G>A | p.? | 67 |
| *4294097* | TP53 | c.731G>A | p.Gly244Asp | 9 |
| *4294097* | TP53 | c.711G>T | p.Met237Ile | 6 |
| *4294097* | TP53 | c.711G>C | p.Met237Ile | 5 |
| *4294097* | SF3B1 | c.2098A>G | p.Lys700Glu | 40 |
| *4294097* | IDH1 | c.394C>T | p.Arg132Cys | 26 |
| *4294404* | TP53 | c.481G>A | p.Ala161Thr | 85 |
| *4294404* | IDH2 | c.419G>A | p.Arg140Gln | 46 |
| *4294638* | U2AF1 | c.470A>C | p.Gln157Pro | 45 |
| *4294638* | ASXL1 | c.1934dupG | p.Gly646Trpfs*12 | 39 |
| *4294638* | IDH2 | c.419G>T | p.Arg140Leu | 49 |
| *4294638* | RUNX1 | c.1102dupA | p.Met368Asnfs*232 | 14 |
| *4294638* | PHF6 | c.245delG | p.Cys82Phefs*15 | 9 |
| *4294638* | PHF6 | c.821G>A | p.Arg274Gln | 73 |
| *4296431* | ASXL1 | c.1934dupG | p.Gly646Trpfs*12 | 35 |
| *4296431* | STAG2 | c.2063T>A | p.Leu688* | 29 |
| *4296431* | RUNX1 | c.958C>T | p.Arg320* | 30 |
| *4296431* | NRAS | c.35G>T | p.Gly12Val | 12 |
| *4296431* | CSF3R | c.1919C>A | p.Thr640Asn | 9 |
| *4298484* | U2AF1 | c.101C>A | p.Ser34Tyr | 31 |
| *4298484* | TET2 | c.4075C>T | p.Arg1359Cys | 49 |
| *4298484* | TET2 | c.5369_5372delTTTC | p.Leu1790Profs*29 | 49 |
| *4298484* | NRAS | c.35G>A | p.Gly12Asp | 5 |
| *4298484* | NRAS | c.34G>A | p.Gly12Ser | 9 |
| *4298484* | NRAS | c.34G>C | p.Gly12Arg | 30 |
| *4300043* | DNMT3A | c.2645G>A | p.Arg882His | 34 |
| *4300043* | CSF3R | c.2278C>A | p.Pro760Thr | 46 |
| *4300771* | U2AF1 | c.470A>C | p.Gln157Pro | 34 |
| *4300771* | ASXL1 | c.1900_1922delAGAGAGGCGGCCACCACTGCCAT | p.Glu635Argfs*15 | 30 |
| *4300771* | DNMT3A | c.2645G>A | p.Arg882His | 34 |
| *4300771* | IDH1 | c.394C>T | p.Arg132Cys | 42 |
| *4300771* | RUNX1 | c.620G>A | p.Arg207Gln | 32 |
| *4301756* | ASXL1 | c.2302C>T | p.Gln768* | 6 |
| *4301756* | JAK2 | c.1849G>T | p.Val617Phe | 4 |
| *4301761* | TP53 | c.725G>A | p.Cys242Tyr | 95 |
| *4303024* | SRSF2 | c.284C>T | p.Pro95Leu | 46 |
| *4303024* | SF3B1 | c.1998G>T | p.Lys666Asn | 43 |
| *4303024* | ASXL1 | c.1934dupG | p.Gly646Trpfs*12 | 36 |
| *4303024* | RUNX1 | c.496C>T | p.Arg166* | 44 |
| *4303403* | DNMT3A | c.2141C>G | p.Ser714Cys | 8 |
| *4310995* | ASXL1 | c.1729delT | p.Ser577Hisfs*126 | 26 |
| *4310995* | ETV6 | c.1057C>T | p.Arg353Trp | 23 |
| *4311309* | ZRSR2 | c.1127G>A | p.Arg376Lys | 99 |
| *4311309* | ASXL1 | c.1934dupG | p.Gly646Trpfs*12 | 33 |
| *4311309* | TET2 | c.3029delA | p.Glu1010Glyfs*23 | 36 |
| *4311309* | TET2 | c.5666C>G | p.Pro1889Arg | 36 |
| *4314134* | TP53 | c.814delG | p.Val272Cysfs*73 | 29 |
| *4314134* | TP53 | c.448_454dupACACCCC | p.Pro152Hisfs*31 | 24 |
| *4314134* | CALR | c.1099_1150del | p.Leu367Thrfs*46 | 15 |
| *4315266* | SRSF2 | c.284C>A | p.Pro95His | 35 |
| *4315266* | STAG2 | c.702delA | p.Leu235* | 41 |
| *4315266* | DNMT3A | c.1811G>A | p.Arg604Gln | 43 |
| *4315266* | IDH2 | c.419G>A | p.Arg140Gln | 46 |
| *4315266* | GATA2 | c.302dupG | p.Lys102Glnfs*83 | 6 |
| *4315266* | FLT3 | c.2503G>C | p.Asp835His | 6 |
| *4315266* | PTPN11 | c.1508G>C | p.Gly503Ala | 11 |
| *4316032* | TP53 | c.743G>A | p.Arg248Gln | 94 |
| *4319561* | EZH2 | c.2044G>A | p.Ala682Thr | 43 |
| *4320751* | WT1 | c.1129_1139dupACTCTTGTACG | p.Ser381Leufs*72 | 17 |
| *4320751* | WT1 | c.1385G>A | p.Arg462Gln | 26 |
| *4323213* | TP53 | c.817C>A | p.Arg273Ser | 93 |
| *4324963* | DNMT3A | c.2248C>T | p.Pro750Ser | 44 |
| *4324963* | JAK2 | c.1849G>T | p.Val617Phe | 14 |
| *4326466* | TP53 | c.768_769delAC | p.Leu257Glyfs*6 | 56 |
| *4329114* | WT1 | c.1141_1144dupTCGG | p.Ala382Valfs*4 | 44 |
| *4329114* | WT1 | c.1106_1107insGGTG | p.Arg370Valfs*16 | 42 |
| *4329164* | TP53 | c.503A>C | p.His168Pro | 52 |
| *4330706* | U2AF1 | c.101C>A | p.Ser34Tyr | 11 |
| *4333090* | SF3B1 | c.1984C>G | p.His662Asp | 23 |
| *4333642* | IDH1 | c.394C>G | p.Arg132Gly | 37 |
| *4345330* | U2AF1 | c.101C>T | p.Ser34Phe | 33 |
| *4345330* | BCOR | c.4174-1G>A | p.? | 77 |
| *4345330* | BCORL1 | c.3796C>T | p.Arg1266* | 13 |
| *4345330* | BCORL1 | c.1268dupC | p.Ala424Cysfs*111 | 27 |
| *4345330* | DNMT3A | c.2644C>T | p.Arg882Cys | 41 |
| *4345330* | TET2 | c.2674C>T | p.Gln892* | 38 |
| *4345330* | KRAS | c.38G>A | p.Gly13Asp | 10 |
| *4347888* | TP53 | c.724T>C | p.Cys242Arg | 86 |
| *4352821* | TP53 | c.375+1G>C | p.? | 60 |
| *4352821* | MPL | c.1544G>T | p.Trp515Leu | 34 |
| *4353516* | ASXL1 | c.2077C>T | p.Arg693* | 37 |
| *4353516* | EZH2 | c.1580C>A | p.Pro527His | 44 |
| *4353516* | EZH2 | c.1654T>C | p.Cys552Arg | 44 |
| *4353516* | IDH1 | c.394C>T | p.Arg132Cys | 24 |
| *4353516* | RUNX1 | c.958C>T | p.Arg320* | 20 |
| *4353516* | IKZF1 | c.476A>G | p.Asn159Ser | 18 |
| *4353516* | JAK2 | c.1849G>T | p.Val617Phe | 45 |
| *4353516* | CBL | c.1151G>T | p.Cys384Phe | 33 |
| *4353591* | SRSF2 | c.284C>A | p.Pro95His | 45 |
| *4353591* | ASXL1 | c.1934dupG | p.Gly646Trpfs*12 | 32 |
| *4353591* | STAG2 | c.661_663dupCTG | p.Leu221dup | 90 |
| *4353591* | IDH2 | c.419G>A | p.Arg140Gln | 49 |
| *4353591* | CEBPA | c.68dupC | p.His24Alafs*84 | 52 |
| *4353614* | TP53 | c.376-1G>A | p.? | 29 |
| *4353614* | TP53 | c.279delG | p.Ser94Hisfs*29 | 34 |
| *4354343* | TP53 | c.724T>G | p.Cys242Gly | 27 |
| *4354343* | BCORL1 | c.5077G>A | p.Glu1693Lys | 99 |
| *4354343* | IDH1 | c.394C>T | p.Arg132Cys | 16 |
| *4354343* | JAK2 | c.1849G>T | p.Val617Phe | 21 |
| *4354440* | SRSF2 | c.284C>A | p.Pro95His | 29 |
| *4354440* | ASXL1 | c.1934dupG | p.Gly646Trpfs*12 | 28 |
| *4354440* | STAG2 | c.3187_3188dupAT | p.Ser1065Alafs*41 | 55 |
| *4354440* | TET2 | c.5152G>T | p.Val1718Leu | 47 |
| *4354440* | IDH2 | c.419G>A | p.Arg140Gln | 6 |
| *4354440* | IDH1 | c.395G>A | p.Arg132His | 17 |
| *4354440* | RUNX1 | c.601C>T | p.Arg201* | 35 |
| *4354440* | NRAS | c.34G>C | p.Gly12Arg | 3 |
| *4354440* | FLT3 | c.1742_1798dup | p.Val581_Tyr599dup | 15 |
| *4359521* | SRSF2 | c.284C>T | p.Pro95Leu | 41 |
| *4359521* | ASXL1 | c.1934dupG | p.Gly646Trpfs*12 | 34 |
| *4359521* | TET2 | c.780delG | p.Leu260Phefs*33 | 59 |
| *4359521* | TET2 | c.1692G>A | p.Trp564* | 49 |
| *4361917* | ASXL1 | c.1934dupG | p.Gly646Trpfs*12 | 32 |
| *4361917* | EZH2 | c.786dupC | p.Asn263Glnfs*8 | 81 |
| *4361917* | STAG2 | c.2857C>T | p.Arg953* | 89 |
| *4361917* | RUNX1 | c.245_246insAGCA | p.Asp84Glyfs*55 | 40 |
| *4361917* | NRAS | c.35G>C | p.Gly12Ala | 5 |
| *4362623* | BCOR | c.1712C>G | p.Ser571* | 13 |
| *4362623* | BCOR | c.4456A>T | p.Lys1486* | 41 |
| *4362623* | EZH2 | c.2022G>C | p.Leu674Phe | 30 |
| *4362623* | DNMT3A | c.2330C>A | p.Pro777His | 22 |
| *4362623* | DNMT3A | c.2645G>A | p.Arg882His | 43 |
| *4362623* | IDH2 | c.515G>A | p.Arg172Lys | 46 |
| *4364329* | U2AF1 | c.470A>C | p.Gln157Pro | 43 |
| *4364329* | IDH1 | c.395G>A | p.Arg132His | 47 |
| *4364329* | NPM1 | c.860_863dupTCTG | p.Trp288Cysfs*12 | 26 |
| *4368006* | TET2 | c.2881G>T | p.Glu961* | 5 |
| *4368006* | TET2 | c.4642C>T | p.Gln1548* | 31 |
| *4368006* | RUNX1 | c.484A>T | p.Arg162Trp | 50 |
| *4368006* | SETBP1 | c.2608G>C | p.Gly870Arg | 5 |
| *4371522* | TP53 | c.403delT | p.Cys135Alafs*35 | 58 |
| *4372429* | TP53 | c.488A>G | p.Tyr163Cys | 24 |
| *4375434* | TP53 | c.733G>A | p.Gly245Ser | 33 |
| *4375434* | TP53 | c.503A>G | p.His168Arg | 34 |
| *4375434* | DNMT3A | c.1314dupC | p.Met439Hisfs*6 | 31 |
| *4375434* | TET2 | c.4242_4246dupGCTTC | p.His1416Argfs*34 | 6 |
| *4375434* | IDH1 | c.394C>T | p.Arg132Cys | 11 |
| *4377103* | TP53 | c.1024C>T | p.Arg342* | 43 |
| *4377103* | TP53 | c.659A>G | p.Tyr220Cys | 38 |
| *4377103* | TET2 | c.1311C>A | p.Tyr437* | 48 |
| *4377103* | TET2 | c.5852G>A | p.Arg1951Gln | 51 |
| *4377103* | BRAF | c.1781A>G | p.Asp594Gly | 42 |
| *4378474* | TP53 | c.574C>T | p.Gln192* | 42 |
| *4378474* | DNMT3A | c.1643T>A | p.Met548Lys | 38 |
| *4379717* | U2AF1 | c.101C>T | p.Ser34Phe | 20 |
| *4379717* | BCOR | c.4936dupC | p.Leu1646Profs*6 | 39 |
| *4379717* | BCORL1 | c.3158A>G | p.Lys1053Arg | 100 |
| *4379717* | RUNX1 | c.497G>A | p.Arg166Gln | 19 |
| *4383834* | BCOR | c.1539delG | p.Pro514Glnfs*14 | 60 |
| *4387237* | U2AF1 | c.470A>G | p.Gln157Arg | 29 |
| *4387237* | ASXL1 | c.2278C>T | p.Gln760* | 29 |
| *4387237* | ASXL1 | c.3745A>G | p.Met1249Val | 47 |
| *4387237* | STAG2 | c.2020C>T | p.Gln674* | 18 |
| *4387237* | TET2 | c.2332C>T | p.Gln778* | 27 |
| *4395325* | NRAS | c.181C>A | p.Gln61Lys | 53 |
| *4395325* | PTPN11 | c.289G>C | p.Glu97Gln | 48 |
| *4396312* | ZRSR2 | c.868C>T | p.Arg290* | 68 |
| *4396312* | BCOR | c.2271C>G | p.Asp757Glu | 99 |
| *4396312* | BCOR | c.1024C>T | p.Arg342* | 70 |
| *4396312* | BCORL1 | c.3994C>T | p.Arg1332* | 78 |
| *4396312* | STAG2 | c.1840C>T | p.Arg614* | 62 |
| *4396312* | DNMT3A | c.2645G>A | p.Arg882His | 36 |
| *4396312* | TET2 | c.3785G>A | p.Arg1262Gln | 41 |
| *4396312* | TET2 | c.4075C>T | p.Arg1359Cys | 38 |
| *4396312* | IDH2 | c.419G>A | p.Arg140Gln | 3 |
| *4396443* | BCOR | c.4123C>T | p.Arg1375Trp | 84 |
| *4396443* | PTPN11 | c.1508G>T | p.Gly503Val | 37 |
| *4402960* | EZH2 | c.2067dupT | p.Arg690Serfs*19 | 69 |
| *4402960* | DNMT3A | c.22G>A | p.Gly8Ser | 34 |
| *4402960* | RUNX1 | c.1036dupC | p.Arg346Profs*254 | 57 |
| *4402960* | GATA2 | c.1234dupG | p.Glu412Glyfs*124 | 64 |
| *4402960* | KIT | c.1249_1255delinsT | p.Thr417_Asp419delinsTyr | 22 |
| *4405480* | TP53 | c.647T>A | p.Val216Glu | 93 |
| *4405480* | RUNX1 | c.179C>T | p.Ala60Val | 64 |
| *4405480* | NOTCH1 | c.5273G>A | p.Arg1758His | 52 |
| *4405632* | IDH1 | c.394C>T | p.Arg132Cys | 36 |
| *4405632* | CEBPA | c.537_541dupCCCTT | p.Tyr181Serfs*139 | 30 |
| *4405632* | PHF6 | c.346C>T | p.Arg116* | 66 |
| *4405632* | MPL | c.1544G>T | p.Trp515Leu | 38 |
| *4408001* | SRSF2 | c.284C>A | p.Pro95His | 7 |
| *4408001* | ASXL1 | c.2866_2867delCT | p.Leu956Tyrfs*13 | 7 |
| *4408001* | STAG2 | c.1304+1G>A | p.? | 10 |
| *4408498* | GATA2 | c.649_653dupCTGAC | p.Glu219* | 45 |
| *4408691* | TP53 | c.659A>G | p.Tyr220Cys | 88 |
| *4408691* | DNMT3A | c.2645G>A | p.Arg882His | 45 |
| *4408691* | TET2 | c.2585dupT | p.Leu862Phefs*10 | 43 |
| *4410456* | BCOR | c.2419_2420dupGA | p.Asp807Glufs*50 | 39 |
| *4410456* | BCORL1 | c.2661delG | p.Gln888Asnfs*37 | 43 |
| *4410456* | TET2 | c.2604T>G | p.Phe868Leu | 41 |
| *4410456* | IDH1 | c.394C>T | p.Arg132Cys | 23 |
| *4410456* | CUX1 | c.3161C>T | p.Ser1054Leu | 45 |
| *4410456* | NRAS | c.38G>A | p.Gly13Asp | 2 |
| *4410456* | NRAS | c.35G>A | p.Gly12Asp | 13 |
| *4410456* | CSF3R | c.2278C>A | p.Pro760Thr | 56 |
| *4412290* | TP53 | c.376-2A>G | p.? | 55 |
| *4413320* | TP53 | c.216dupC | p.Val73Argfs*76 | 60 |
| *4413320* | SRSF2 | c.349C>T | p.Arg117Cys | 48 |
| *4413320* | CALR | c.1099_1150del | p.Leu367Thrfs*46 | 59 |
| *4413812* | TP53 | c.783-1G>A | p.? | 14 |
| *4413812* | DNMT3A | c.2645G>A | p.Arg882His | 15 |
| *4413812* | DNMT3A | c.1969G>A | p.Val657Met | 23 |
| *4416944* | TP53 | c.993+1G>A | p.? | 43 |
| *4416944* | TP53 | c.799C>T | p.Arg267Trp | 44 |
| *4417609* | NRAS | c.35G>A | p.Gly12Asp | 4 |
| *4420607* | SRSF2 | c.284C>G | p.Pro95Arg | 39 |
| *4420607* | TET2 | c.3735C>G | p.Tyr1245* | 36 |
| *4420607* | TET2 | c.3782G>A | p.Arg1261His | 32 |
| *4420651* | U2AF1 | c.470A>C | p.Gln157Pro | 26 |
| *4420651* | ASXL1 | c.1851dupT | p.Lys618* | 27 |
| *4420651* | STAG2 | c.367C>T | p.Gln123* | 47 |
| *4420651* | RAD21 | c.1242T>G | p.Asp414Glu | 48 |
| *4420651* | DNMT3A | c.2644C>T | p.Arg882Cys | 28 |
| *4420651* | IDH2 | c.419G>A | p.Arg140Gln | 3 |
| *4420651* | RUNX1 | c.268dupG | p.Val90Glyfs*48 | 22 |
| *4433296* | TP53 | c.659A>G | p.Tyr220Cys | 37 |
| *4433296* | TP53 | c.102delC | p.Leu35Cysfs*9 | 38 |
| *4433296* | DNMT3A | c.1510delC | p.Leu504Trpfs*147 | 39 |
| *4434373* | RUNX1 | c.460C>T | p.Gln154* | 42 |
| *4437213* | TP53 | c.527G>A | p.Cys176Tyr | 28 |
| *4440994* | TP53 | c.536A>G | p.His179Arg | 65 |
| *4440994* | IDH1 | c.394C>T | p.Arg132Cys | 38 |
| *4440994* | KIT | c.1588G>A | p.Val530Ile | 49 |
| *4454139* | SRSF2 | c.284C>A | p.Pro95His | 34 |
| *4454139* | ASXL1 | c.2091delG | p.Pro698Argfs*5 | 36 |
| *4454139* | STAG2 | c.2316dupT | p.Gln773Serfs*12 | 32 |
| *4454139* | IDH2 | c.419G>A | p.Arg140Gln | 32 |
| *4454139* | CUX1 | c.415G>T | p.Glu139* | 30 |
| *4455392* | DNMT3A | c.2645G>A | p.Arg882His | 23 |
| *4455392* | TET2 | c.685dupA | p.Thr229Asnfs*25 | 39 |
| *4455392* | NPM1 | c.863_864insCACT | p.Trp288Cysfs*12 | 22 |
| *4464369* | TP53 | c.112C>T | p.Gln38* | 45 |
| *4464369* | TP53 | c.455C>T | p.Pro152Leu | 44 |
| *4465829* | RAD21 | c.372dupA | p.Asp125Argfs*2 | 44 |
| *4465829* | DNMT3A | c.915G>A | p.Trp305* | 47 |
| *4465829* | NPM1 | c.860_863dupTCTG | p.Trp288Cysfs*12 | 32 |
| *4465836* | TP53 | c.514G>T | p.Val172Phe | 98 |
| *4465836* | TET2 | c.5602C>G | p.His1868Asp | 47 |
| *4465836* | CEBPA | c.961A>G | p.Asn321Asp | 97 |
| *4465836* | JAK2 | c.1849G>T | p.Val617Phe | 48 |
| *4474162* | TP53 | c.817C>T | p.Arg273Cys | 86 |
| *4474173* | TP53 | c.560-1G>A | p.? | 66 |
| *4474173* | BCOR | c.1475A>G | p.Asn492Ser | 48 |
| *4478015* | SF3B1 | c.1876A>G | p.Asn626Asp | 15 |
| *4478015* | TET2 | c.2862G>A | p.Trp954* | 6 |
| *4480428* | TET2 | c.3467delA | p.Asn1156Metfs*70 | 79 |
| *4490611* | TP53 | c.102delC | p.Leu35Cysfs*9 | 50 |
| *4494470* | DNMT3A | c.89A>C | p.Glu30Ala | 49 |
| *4494470* | DNMT3A | c.1816C>T | p.Gln606* | 20 |
| *4494470* | IDH1 | c.395G>T | p.Arg132Leu | 11 |
| *4494470* | PHF6 | c.374+2T>A | p.? | 11 |
| *4494470* | MYD88 | c.649G>T | p.Val217Phe | 11 |
| *4494965* | TP53 | c.565delG | p.Ala189Profs*58 | 68 |
| *4494965* | BCOR | c.2609C>G | p.Thr870Ser | 47 |
| *4495718* | U2AF1 | c.101C>T | p.Ser34Phe | 45 |
| *4495718* | TET2 | c.3805A>T | p.Arg1269* | 45 |
| *4495718* | TET2 | c.4594C>T | p.Gln1532* | 48 |
| *4499017* | SF3B1 | c.2098A>G | p.Lys700Glu | 42 |
| *4499017* | TET2 | c.4600C>T | p.Gln1534* | 40 |
| *4499017* | NRAS | c.182A>T | p.Gln61Leu | 10 |
| *4500230* | SRSF2 | c.284C>G | p.Pro95Arg | 47 |
| *4500230* | ASXL1 | c.2144delG | p.Arg715Lysfs*10 | 41 |
| *4500230* | RAD21 | c.127_128delAG | p.Ser43Tyrfs*36 | 27 |
| *4500230* | TET2 | c.4457C>G | p.Ser1486* | 51 |
| *4500230* | TET2 | c.565delA | p.Ser189Valfs*18 | 37 |
| *4508994* | PTPN11 | c.1504T>C | p.Ser502Pro | 39 |
| *4524081* | TP53 | c.844C>T | p.Arg282Trp | 44 |
| *4524081* | TP53 | c.659A>G | p.Tyr220Cys | 43 |
| *4524299* | ASXL1 | c.1934dupG | p.Gly646Trpfs*12 | 31 |
| *4524299* | STAG2 | c.3133C>T | p.Arg1045* | 44 |
| *4524299* | DNMT3A | c.2644C>T | p.Arg882Cys | 46 |
| *4524299* | IDH2 | c.419G>A | p.Arg140Gln | 43 |
| *4524299* | NRAS | c.35G>A | p.Gly12Asp | 31 |
| *4524299* | KIT | c.1588G>A | p.Val530Ile | 50 |
| *4536180* | SF3B1 | c.1984C>G | p.His662Asp | 30 |
| *4536180* | ASXL1 | c.2740G>T | p.Glu914* | 32 |
| *4536180* | DNMT3A | c.2644C>T | p.Arg882Cys | 34 |
| *4536180* | IDH1 | c.394C>T | p.Arg132Cys | 18 |
| *4536180* | RUNX1 | c.958C>T | p.Arg320* | 17 |
| *4545342* | TP53 | c.754_762delCTCACCATC | p.Leu252_Ile254del | 21 |
| *4545342* | TP53 | c.466C>G | p.Arg156Gly | 18 |
| *4547917* | SF3B1 | c.1997A>T | p.Lys666Met | 49 |
| *4547917* | RUNX1 | c.660delC | p.Ser222Profs*15 | 43 |
| *4547917* | GATA2 | c.920G>T | p.Arg307Leu | 38 |
| *4549852* | SRSF2 | c.284C>T | p.Pro95Leu | 48 |
| *4549852* | ASXL1 | c.1900_1922delAGAGAGGCGGCCACCACTGCCAT | p.Glu635Argfs*15 | 48 |
| *4549852* | IDH2 | c.419G>A | p.Arg140Gln | 44 |
| *4549852* | FLT3 | c.1776_1811dup | p.Trp603_Glu604ins12 | 10 |
| *4549852* | KRAS | c.38G>A | p.Gly13Asp | 20 |
| *4557040* | SRSF2 | c.284_307delCCCCGGACTCACACCACAGCCGCC | p.Pro95_Arg102del | 50 |
| *4557040* | ASXL1 | c.1762C>T | p.Gln588* | 47 |
| *4557040* | RUNX1 | c.613+1G>A | p.? | 45 |
| *4557040* | ETV6 | c.412_413dupAA | p.Asn138Lysfs*72 | 42 |
| *4567029* | BCORL1 | c.5042dupC | p.Gly1682Argfs*4 | 29 |
| *4567029* | KMT2A | c.1701_2576dup | p.Pro568_Ser859dup | 27 |
| *4567029* | DNMT3A | c.1741T>C | p.Trp581Arg | 45 |
| *4567029* | IDH1 | c.394C>T | p.Arg132Cys | 48 |
| *4567029* | CEBPA | c.747_750delTGGC | p.Gly250Alafs*67 | 49 |
| *4567472* | TP53 | c.659A>G | p.Tyr220Cys | 36 |
| *4567472* | TP53 | c.224dupC | p.Ala76Cysfs*73 | 35 |
| *4567472* | ETV6 | c.556A>G | p.Ile186Val | 47 |
| *4569528* | TP53 | c.842A>G | p.Asp281Gly | 61 |
| *4569528* | RUNX1 | c.155T>A | p.Met52Lys | 50 |
| *4573308* | TP53 | c.830G>A | p.Cys277Tyr | 13 |
| *4573308* | CUX1 | c.4448G>A | p.Arg1483His | 50 |
| *4573308* | CUX1 | c.3015delA | p.Gly1006Alafs*12 | 19 |
| *4573308* | CUX1 | c.3826C>T | p.Gln1276* | 12 |
| *4575259* | BRAF | c.1799T>A | p.Val600Glu | 6 |
| *4576677* | TP53 | c.780_782+5delinsGACCT | p.? | 36 |
| *4576677* | STAG2 | c.536T>C | p.Ile179Thr | 37 |
| *4589822* | TP53 | c.430C>T | p.Gln144* | 67 |
| *4591772* | TP53 | c.713G>A | p.Cys238Tyr | 89 |
| *4591772* | DNMT3A | c.1933A>G | p.Thr645Ala | 45 |
| *4591772* | PTPN11 | c.182A>T | p.Asp61Val | 34 |
| *4595042* | CBL | c.1197_1431+159del | p.? | 17 |
| *4598664* | TP53 | c.752T>G | p.Ile251Ser | 90 |
| *4598664* | BCOR | c.934C>T | p.Gln312* | 39 |
| *4598664* | KMT2A | c.6572G>A | p.Arg2191Gln | 33 |
| *4599321* | TP53 | c.646G>A | p.Val216Met | 88 |
| *4601661* | TP53 | c.776A>T | p.Asp259Val | 18 |
| *4601661* | EZH2 | c.638G>A | p.Arg213His | 45 |
| *4601661* | DNMT3A | c.2645G>A | p.Arg882His | 42 |
| *4601661* | JAK2 | c.1849G>T | p.Val617Phe | 7 |
| *4604634* | BCOR | c.3649C>T | p.Arg1217* | 82 |
| *4604634* | KMT2A | c.7070C>G | p.Pro2357Arg | 52 |
| *4604634* | DNMT3A | c.2196T>G | p.Phe732Leu | 85 |
| *4604634* | IDH2 | c.419G>A | p.Arg140Gln | 46 |
| *4604634* | ETV6 | c.196G>A | p.Val66Ile | 51 |
| *4604634* | NOTCH1 | c.6782G>A | p.Gly2261Asp | 52 |
| *4604634* | NRAS | c.38G>A | p.Gly13Asp | 20 |
| *4604634* | CSF3R | c.2326C>T | p.Gln776* | 11 |
| *4604857* | DNMT3A | c.2523G>C | p.Lys841Asn | 44 |
| *4604857* | FLT3 | c.1802_1803ins24 | p.Lys602_Trp603ins8 | 18 |
| *4604857* | NPM1 | c.860_863dupTCTG | p.Trp288Cysfs*12 | 42 |
| *4606272* | U2AF1 | c.471G>C | p.Gln157His | 37 |
| *4606272* | ASXL1 | c.1900_1922delAGAGAGGCGGCCACCACTGCCAT | p.Glu635Argfs*15 | 37 |
| *4606272* | DNMT3A | c.447G>A | p.Ala149= | 50 |
| *4606272* | GATA2 | c.844delC | p.Gln282Serfs*44 | 36 |
| *4606272* | CUX1 | c.1820G>A | p.Trp607* | 37 |
| *4606272* | PHF6 | c.812A>G | p.Glu271Gly | 35 |
| *4607860* | TP53 | c.701A>G | p.Tyr234Cys | 74 |
| *4607860* | TP53 | c.97-1G>C | p.? | 8 |
| *4615929* | TP53 | c.407A>C | p.Gln136Pro | 56 |
| *4615929* | BCOR | c.3490C>T | p.Arg1164* | 6 |
| *4619366* | ASXL1 | c.3015delT | p.Phe1005Leufs*19 | 49 |
| *4619366* | DNMT3A | c.2117G>A | p.Gly706Glu | 50 |
| *4619366* | CUX1 | c.3317C>T | p.Pro1106Leu | 19 |
| *4619366* | PTPN11 | c.1508G>A | p.Gly503Glu | 46 |
| *4619366* | SETBP1 | c.2602G>A | p.Asp868Asn | 51 |
| *4619794* | U2AF1 | c.467G>A | p.Arg156His | 46 |
| *4619794* | BCOR | c.3239-1G>A | p.? | 93 |
| *4619794* | STAG2 | c.1028dupT | p.Arg344Lysfs*14 | 91 |
| *4619794* | DNMT3A | c.2645G>A | p.Arg882His | 42 |
| *4619794* | GATA2 | c.1075T>G | p.Leu359Val | 49 |
| *4619794* | NRAS | c.38G>A | p.Gly13Asp | 28 |
| *4619794* | NRAS | c.37G>C | p.Gly13Arg | 6 |
| *4619794* | KRAS | c.35G>A | p.Gly12Asp | 15 |
| *4622058* | EZH2 | c.2069G>A | p.Arg690His | 14 |
| *4622058* | DNMT3A | c.2645G>C | p.Arg882Pro | 49 |
| *4622058* | IDH2 | c.419G>A | p.Arg140Gln | 48 |
| *4622058* | RUNX1 | c.592G>A | p.Asp198Asn | 48 |
| *4622058* | FLT3 | c.1837_1837+1ins54 | p.? | 10 |
| *4625561* | NRAS | c.35G>T | p.Gly12Val | 17 |
| *4625561* | KRAS | c.35G>A | p.Gly12Asp | 5 |
| *4628553* | DNMT3A | c.2194T>A | p.Phe732Ile | 45 |
| *4628553* | IDH2 | c.419G>A | p.Arg140Gln | 9 |
| *4628553* | CUX1 | c.3632A>G | p.Asp1211Gly | 51 |
| *4628553* | FLT3 | c.1790_1837dup | p.Phe612_Gly613ins16 | 17 |
| *4628553* | CBL | c.1149A>G | p.Ile383Met | 58 |
| *4628553* | NPM1 | c.860_863dupTCTG | p.Trp288Cysfs*12 | 43 |
| *4645115* | EZH2 | c.149T>C | p.Leu50Ser | 78 |
| *4645115* | ETV6 | c.1138T>G | p.Trp380Gly | 38 |
| *4651306* | SRSF2 | c.284C>G | p.Pro95Arg | 6 |
| *4651306* | BCOR | c.1779dupC | p.Val594Argfs*17 | 77 |
| *4651306* | DNMT3A | c.2645G>A | p.Arg882His | 43 |
| *4651306* | TET2 | c.5583delA | p.Val1862Trpfs*25 | 10 |
| *4651306* | RUNX1 | c.929_934delinsCCTT | p.Met310Thrfs*289 | 31 |
| *4665121* | SF3B1 | c.2098A>G | p.Lys700Glu | 49 |
| *4665121* | STAG2 | c.2455dupT | p.Ser819Phefs*6 | 92 |
| *4665121* | FLT3 | c.2503G>T | p.Asp835Tyr | 42 |
| *4665121* | SETBP1 | c.584C>T | p.Thr195Met | 50 |
| *4665121* | NPM1 | c.863_864insCCTG | p.Trp288Cysfs*12 | 38 |
| *4667870* | SRSF2 | c.284C>A | p.Pro95His | 49 |
| *4667870* | ASXL1 | c.1900_1922delAGAGAGGCGGCCACCACTGCCAT | p.Glu635Argfs*15 | 58 |
| *4667870* | STAG2 | c.793_794insGA | p.Glu265Glyfs*18 | 82 |
| *4667870* | IDH2 | c.419G>A | p.Arg140Gln | 44 |
| *4667870* | NRAS | c.34G>A | p.Gly12Ser | 41 |
| *4670639* | TET2 | c.4187_4188delinsAT | p.Cys1396Tyr | 40 |
| *4670639* | IDH2 | c.419G>A | p.Arg140Gln | 44 |
| *4670639* | NPM1 | c.860_863dupTCTG | p.Trp288Cysfs*12 | 36 |
| *4687271* | TP53 | c.725G>A | p.Cys242Tyr | 85 |
| *4687271* | CUX1 | c.295G>A | p.Val99Ile | 6 |
| *4707468* | TP53 | c.216dupC | p.Val73Argfs*76 | 38 |
| *5981010* | DNMT3A | c.2645G>A | p.Arg882His | 12 |
| *5981010* | TET2 | c.1873A>G | p.Thr625Ala | 51 |
| *5981010* | TET2 | c.100C>T | p.Leu34Phe | 51 |
| *5981010* | IDH2 | c.515G>A | p.Arg172Lys | 10 |
| *5981010* | PHF6 | c.902_903insA | FS | 22 |
| *6210996* | SRSF2 | c.284C>A | p.Pro95His | 35 |
| *6210996* | ASXL1 | c.1934dupG | p.Gly646Trpfs*12 | 27 |
| *6210996* | STAG2 | c.1304+2T>C | p.? | 39 |
| *6210996* | STAG2 | c.2314T>C | p.Cys772Arg | 54 |
| *6210996* | STAG2 | c.3467+1G>A | p.? | 11 |
| *6210996* | IDH2 | c.419G>A | p.Arg140Gln | 31 |
| *6271510* | SRSF2 | c.284C>A | p.Pro95His | 45 |
| *6271510* | ASXL1 | c.1934dupG | p.Gly646Trpfs*12 | 37 |
| *6271510* | DNMT3A | c.89A>C | p.Glu30Ala | 55 |
| *6271510* | RUNX1 | c.793_794delAG | p.Gln266Aspfs*333 | 18 |
| *6271510* | NRAS | c.34G>A | p.Gly12Ser | 18 |
| *6271510* | FLT3 | c.1715A>G | p.Tyr572Cys | 16 |
